# The three-way switch operation of Rac1/RhoA GTPase-based circuit controlling amoeboid-hybrid-mesenchymal transition

**DOI:** 10.1038/srep06449

**Published:** 2014-09-23

**Authors:** Bin Huang, Mingyang Lu, Mohit Kumar Jolly, Ilan Tsarfaty, José Onuchic, Eshel Ben-Jacob

**Affiliations:** 1 Center for Theoretical Biological Physics, Rice University, Houston, TX 77005-1827, USA; 2 Department of Chemistry, Rice University, Houston, TX 77005-1827, USA; 3 Department of Bioengineering, Rice University, Houston, TX 77005-1827, USA; 4 Department of Physics and Astronomy, Rice University, Houston, TX 77005-1827, USA; 5 Department of Clinical Microbiology and Immunology, Sackler School of Medicine; 6 Department of Biosciences, Rice University, Houston, TX 77005-1827, USA; 7 School of Physics and Astronomy and The Sagol School of Neuroscience, Tel-Aviv University, Tel-Aviv 69978, Israel

## Abstract

Metastatic carcinoma cells exhibit at least two different phenotypes of motility and invasion - amoeboid and mesenchymal. This plasticity poses a major clinical challenge for treating metastasis, while its underlying mechanisms remain enigmatic. Transitions between these phenotypes are mediated by the Rac1/RhoA circuit that responds to external signals such as HGF/SF via c-MET pathway. Using detailed modeling of GTPase-based regulation to study the Rac1/RhoA circuit's dynamics, we found that it can operate as a three-way switch. We propose to associate the circuit's three possible states to the amoeboid, mesenchymal and amoeboid/mesenchymal hybrid phenotype. In particular, we investigated the range of existence of, and the transition between, the three states (phenotypes) in response to Grb2 and Gab1 - two downstream adaptors of c-MET. The results help to explain the regulation of metastatic cells by c-MET pathway and hence can contribute to the assessment of possible clinical interventions.

## Introduction

**Figure 1:**
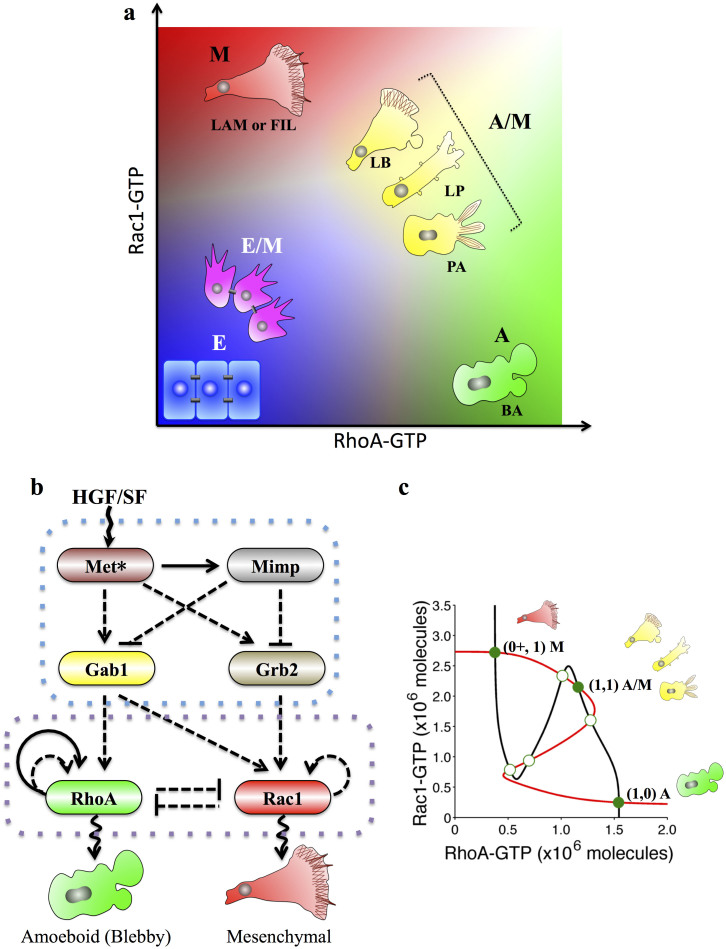
Plasticity of cell migration phenotypes and the core regulatory circuit. (a) The phase diagram showing the Relationship between the activities of the GTPases and the plasticity of cell migration phenotypes. Background colors correspond to the different level of Rac1-GTP and RhoA-GTP - red for high level of Rac1-GTP, green for high level of RhoA-GTP, yellow for high levels of both of them, and blue for low levels of both of them. The activity of the GTPases is hypothetically associated with the cell morphology and mobility. The phenotypes are depicted as cartoons displaying their corresponding morphological features - epithelial phenotype (E), hybrid epithelial-mesenchymal phenotype (E/M), mesenchymal phenotype (M), amoeboid phenotype (A), and hybrid amoeboid-mesenchymal phenotype (A/M). M phenotype is characterized with lamellopodia (LAM) and/or filopodia (FIL), and A phenotype here is specifically referred to Blebby amoeboid (BA) phenotype, which is characterized by blebbing. The A/M phenotype is considered as a set of different morphologies - Lamellipoida with blebs (LB), Lobopodia (LP) and Pseudopodal amoeboid (PA). The blue color is also associated with strong cell-cell adhesion, as observed in E or E/M phenotypes, while the rest colors are associated with single cell migration modes. (b) The core AMT/MAT regulatory circuit connected with the c-MET pathway. The AMT/MAT is mainly regulated by the Rac1/RhoA regulatory circuit, while RhoA and Rac1 are regulated via Grb2 and Gab1 by the c-MET pathway, which receives the external signal from HGF/SF. A solid arrow denotes activation, and a solid bar stands for repression. A solid line represents transcriptional regulation, and a dashed line is for non-transcriptional regulations such as GTP loading. (c) Dynamical system characteristics of the Rac1/RhoA regulatory circuit. The plot shows the nullclines and possible steady states corresponding to Equation (1). Without any external signal (Grb2 = 0, Gab1 = 0), the circuit can be tristable. Red nullcline is for *d*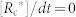 and black nullcline is for 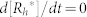. Green solid circles denote the stable fixed points, and green hollow circles denote the unstable fixed points. Each stable point can be associated with a cell phenotype, depicted as a cartoon beside them.

Metastasis is a hallmark of cancer^1^, and responsible for more than 90% of solid tumor deaths^2^. Carcinoma is the most common type of solid tumor, whose metastasis is a complex process that begins when some epithelial cells from the primary tumor undergo epithelial-to-mesenchymal transition (EMT) to lose cell-cell adhesion and gain migratory and invasive mesenchymal characteristics. Carcinoma cells can deploy different strategies (phenotypes) to tread through the extra-cellular matrix (ECM)^3^, and different interactions with the stroma or local microenvironment^1^ (Fig. 1a). Our previous theoretical investigations revealed that the core regulatory circuit of EMT operates as a three-way switch, allowing not only for epithelial (E) and mesenchymal (M) phenotypes but also for a hybrid epithelial/mesenchymal phenotype (E/M), which is associated with collective cell migration^4^,^5^. Yet, the plasticity of the individual cell selection between different motility characteristics (phenotypes) still remained illusive. It is widely accepted that deciphering the underlying mechanisms of cellular plasticity during metastatic invasion is central for designing therapeutic targeting of carcinomas^3^. To help meet this challenge, we present here theoretical investigations of the GTPase-based operation principles of the Rac1/RhoA circuit - the key regulator for amoeboid-to-mesenchymal transition (AMT). The modeling challenge is to simplify the complexity of this circuit whose dynamics involves transcription, translation, and post-translation (GTPase) regulations.

### Phenotypic Plasticity of Individual Cell Migration

Carcinoma cells typically adopt two different phenotypes to invade the three-dimensional (3D) matrix environment - the mesenchymal (M) or the amoeboid (A) (here we specifically refer to blebby amoeboid (BA))^6^ (Fig. 1a). Cells of the M phenotype are elongated and spindle-shaped with their leading edge characterized by lamellopodia (LAM) and/or filopodia (FIL). These cells are able to remodel and even degrade the ECM by secreting Matrix Metalloproteinases (MMPs) and therefore act as ‘path generators’^6^,^7^. Conversely, rather than secreting MMPs to remodel the ECM, cells that exhibit the A phenotype have higher shape plasticity that enables them to squeeze into the gaps in the ECM, thus acting as ‘path finders’^6^,^7^. In 3D environment, many carcinoma cells exhibit mesenchymal-to-amoeboid transition (MAT) and amoeboid-to-mesenchymal transition (AMT) either spontaneously^8^,^9^ or in response to external signals from the microenvironment^6^,^10^,^11^.

In recent studies, carcinoma cells have been observed to have phenotypes with hybrid amoeboid/mesenchymal (A/M) characteristics (Fig. 1a)^8^,^9^,^12^,^13^,^14^,^15^,^16^. For instance, some carcinosarcoma cells have both lamellopodia/filopodia and blebs structures (LB)^8^,^16^, which have also been seen *in vivo* during early development of the developing Fundus^17^ and zebrafish embryos^18^. Some fibroblasts adopt cylindrical-shaped lobopodia phenotype (LP) in the 3D environment with morphological features of both lateral blebs and blunt protrusions^13^, and some leukocytes and neutrophils show pseudopodal amoeboid (PA) migration phenotype in 3D as well as 2D environments with dynamic protrusions in the front part and high contractility in the rear part^12^,^15^. Such a rich phenotypic plasticity during the migration of individual cell enables the tumor cells to adapt to their changing microenvironments, and plays a crucial role in cancer dissemination^19^.

### The Rac1/RhoA GTPase-based Regulatory Circuit

The choice among the aforementioned phenotypes is operated by the Rac1/RhoA regulatory circuit. Rac1 and RhoA belong to the Rho family of small GTPases and act as molecular switches by changing between their active (the GTP-bound state) and inactive (the GDP-bound state and the GDI-bound state) forms^20^. This switching process is regulated by three sets of proteins: GEFs (Guanine Nucleotide Exchange Factors) that catalyze the exchange of bound GDP for GTP, thus elevating the levels of the active GTPases; GAPs (GTPase-activating proteins) that promote the intrinsic GTP hydrolysis rates, thus reducing the concentration of the active GTPases; and GDIs (Guanine nucleotide dissociation inhibitors) that sequester GTPases from the membrane to the cytosol and stabilize the proteins by preventing degradation^21^.

Rac1 and RhoA regulate the phenotypic transitions by controlling the actin polymerization and actomyosin contraction^22^. Therefore, the activities of these two GTPases have been observed to correlate with cell morphology and motility. For example, actomyosin contractility increases in response to the RhoA activation, thus resulting in membrane blebbing and facilitating the amoeboid phenotype (A)^9^,^23^,^24^. On the other hand, the activation of Rac1 results in the formation of focal adhesions and actin polymerization, which leads to the formation of lamellopodia and enables a mesenchymal phenotype (M)^14^,^23^,^25^. Appropriate changes in the relative strengths of these two driving forces (actomyosin contraction vs. actin polymerization) allow for not only the transitions between the A and M phenotypes, but also might enable the transition into/from the hybrid A/M phenotype^8^. These transitions can be triggered by extracellular signals such as Hepatocyte Growth Factor/Scatter Factor (HGF/SF) (Fig. 1b) through the c-MET pathway^26^. c-MET, the specific tyrosine kinase receptor for HGF/SF, is often overexpressed in many carcinomas and correlates with poor patient survival^27^,^28^. The activated c-MET recruits Grb2 (Growth-factor receptor bound protein 2) and Gab1 (Grb2 associated binding protein 1) to regulate the activity of both RhoA and Rac1 (See Supplementary Table S1 and Supplementary Fig. S1 for the detailed molecular interactions). It is contradictory that the HGF/SF/c-MET pathway has been reported to be able to induce either mesenchymal^29^,^30^ or amoeboid^31^ phenotype, therefore the underlying mechanisms of the HGF/SF/c-MET pathway remain elusive.

Importantly, the active GTP-bound forms of Rac1 (RhoA) inhibit the activation of RhoA (Rac1) by promoting the hydrolysis of the active GTP-bound forms of RhoA (Rac1) (See Supplementary Table S1), thus acting as a mutually inhibitory feedback circuit, often named as a toggle switch. The Rac1/RhoA feedback circuit may have unique dynamical properties, because the regulation of the circuit is post-translational in nature, which is distinct from the dynamics of either transcriptional (TF-TF) or transcriptional and translational (miR-TF) regulation in well-studied canonical toggle switches^4^,^5^,^32^,^33^.

While the RhoA-Rac1 circuit has been extensively studied experimentally^9^,^14^,^23^,^24^,^25^,^34^, it has received limited theoretical attention, primarily due to the special small GTPase-based regulation. Here, we developed a new framework to model the small GTPase-based regulation in the context of the Rac1/RhoA circuit by considering detailed transitions among different states of GTPases. We note that this article considers the expression and overall activity level of the GTPases. More specifically, in this first step model, we did not include spatial effects of the GTPases – the fact that Rac1 and RhoA are activated in different spatial compartments of the cell. Inclusion of the spatial effects is important and will be done in future extension of the current model. The model simulations revealed that the Rac1-RhoA circuit can act, for a wide range of realistic parameters, as a three-way (ternary) switch between three possible states: the amoeboid phenotype (A), the mesenchymal phenotype (M) and the hybrid amoeboid/mesenchymal phenotype (A/M). The model demonstrated that Grb2 and Gab1 could induce the activation of both Rac1 and RhoA, which is expected to promote the migration of cells through mesenchymal and amoeboid phenotypes respectively. Since both Grb2 and Gab1 are regulated by the c-MET pathway, this may possibly explain the observation in which HGF/SF/c-MET pathway induces either of these two mutually exclusive phenotypes in different experiments^29^,^30^,^31^. We will discuss several experimental observations that are consistent with our new model. The model provides the first step towards understanding how different levels of the small GTPases, RhoA and Rac1, in a cell govern phenotypic plasticity during carcinoma metastasis under the influence of external signals in the tumor microenvironment.

### General View of the Core Regulatory Unit

For clarity, we will first summarize the key findings of our model on Rac1/RhoA circuit, followed by more detailed presentation of the analysis. In Fig. 1a, we have shown a spectrum of motility/invasion phenotypes during carcinoma cell metastasis. The epithelial (E) and epithelial/mesenchymal hybrid (E/M) phenotypes are not discussed in this article, since we focus on the motility stage that the cells have already undergone complete EMT. The core AMT/MAT regulatory unit, as shown in Fig. 1b, is composed of mutual inhibitions between the active forms of RhoA and Rac1 (RhoA-GTP and Rac1-GTP). This mutually inhibitory circuit is driven by signals from the c-MET pathway through Gab1 and Grb2 (See Supplementary Table S1).

We first considered the stand-alone dynamics of the Rac1/RhoA circuit. We showed that the circuit acts as a three-way switch for a wide range of biologically realistic parameters (Fig. 1c) similar to the characteristic behavior of other ‘self-activating toggle switches’ (SATS)^32^. In the absence of any external signal, the three states of this circuit (Fig. 1c) are: 1. (1, 0) state - high RhoA-GTP with low Rac1-GTP that corresponds to A phenotype, as characterized by blebs (BA). Notably, in some cases, high RhoA-GTP is reported to inhibit cell migration depending on ECM stiffness and cell type^35^,^36^; 2. (0+, 1) state - low RhoA-GTP with high Rac1-GTP that corresponds to M phenotype as identified by the presence of lamellopodia (LAM) and/or filopodia (FIL). Here, we denoted the level of RhoA-GTP as “0+” to take into account the fact that mesenchymal cells still need some active RhoA in the rear part for retraction^37^, while active Rac1 is present at the leading edge. Therefore, in mesenchymal phenotype, the expression levels of the active forms of RhoA and Rac1 are spatially separated^38^, whereas in amoeboid phenotype, active RhoA is almost uniformly distributed^39^; 3. (1, 1) state - balanced relatively high RhoA-GTP and Rac1-GTP that we propose to be consistent with the hybrid A/M phenotypes observed experimentally^8^,^9^,^12^,^13^,^15^,^16^,^17^,^18^. The existence of the hybrid (1, 1) state indicates richer migration plasticity. The predicted multistability in this circuit, i.e. the existence of diverse possible states, can explain the observed existence of the diverse phenotypes during cell migration.

### Effective Model of the Small GTPase-based Regulatory Circuit

**Figure 2:**
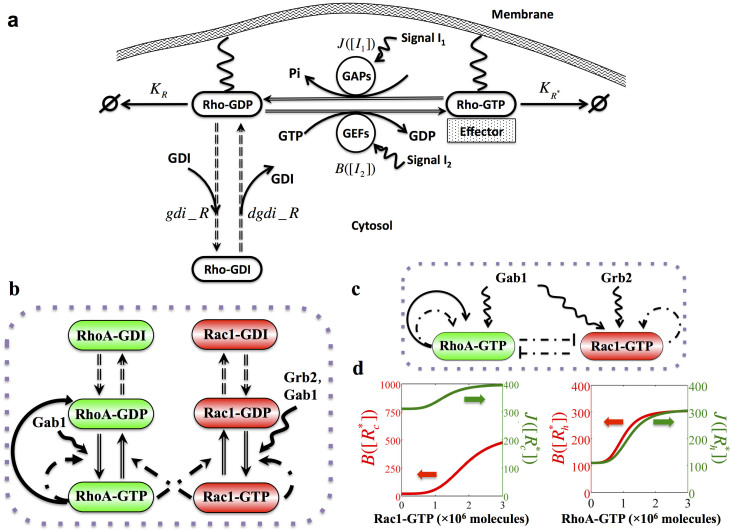
Schematic diagram for theoretical framework and its application on Rac1-RhoA regulatory circuit. (a) Schematic diagram of the regulation of a typical Rho family GTPase (denoted as Rho). The inactive GDP-bound state of Rho (Rho-GDP) and the active GTP-bound state of Rho (Rho-GTP) both bind to the membrane. They can interconvert through the regulations of GAPs (at rate *J*[*I*_1_]) or GEFs (at rate *B*[*I*_2_]), which may be activated by some external input signals (I_1_ and I_2_). Rho-GDP can be released from the membrane by binding to a GDI molecule (at rate *gdi*_*R*) and revert to its membrane-bound state by releasing GDI (at rate *dgdi*_*R*). Rho-GDP and Rho-GTP degrade at rate *K_R_* and 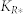 respectively, while the degradation of Rho-GDI was not considered, because GDI binding can stabilize the Rho protein^21^. (b) The RhoA-Rac1 regulatory circuit (Details in Supplementary Table S1). The GTP-bound states of RhoA or Rac1 can promote GTP loading of its own, and meanwhile activate the GTP hydrolysis of the other. RhoA-GTP is also transcriptionally self-activated. Grb2 induces the GTP loading of Rac1, while Gab1 induces that of both Rac1 and RhoA. (c) The effective (reduced Rac1/RhoA) circuit. In terms of Rac1-GTP and Rho-GTP, their mutual inhibitions form a non-canonical toggle switch with positive auto-regulations. A solid arrow denotes activation, a solid bar indicates repression, and the wavy line represents regulation by external signals. The solid double line represents the GTP loading or hydrolysis process while the dashed double one represents the binding or unbinding process of GDI molecules. The dashed-dot lines indicate the indirect regulations on GTP loading or hydrolysis process via GEFs or GAPs. (d) Typical values of the B and the J functions with respect to the concentrations of the GTPases. The B and J functions represent the GTP loading and hydrolysis rates of both Rac1 and RhoA respectively. Both functions increase with the level of GTP-bound Rac1 and RhoA. The parameters for the two functions were listed in Supplementary Table S2.

The challenge posed in modeling the small GTPase-based regulatory circuit is to incorporate the elaborate transitions between different forms of the GTPases. Typically, a GTPase protein can switch among its active (GTP-bound state) and inactive (GDP-bound state and GDI-bound state) forms under the regulation of three sets of proteins (GEFs, GAPs and GDIs)^20^. To understand these features of the GTPases, we developed the theoretical framework for GTPase-based circuit by specifically modeling the detailed molecular interactions as illustrated in Fig. 2a. We utilized the approach to investigate the dynamics of the core Rac1/RhoA regulatory circuit, as shown in Fig. 2b.

The deterministic dynamics of the circuit could be modeled by a set of six chemical rate equations presented in the Supplementary Equations (7) and (8). Yet under the assumption that the total level of Rac1 or RhoA (the sum of levels of GTP-bound, GDP-bound and GDI-bounded form) always reaches a steady state, the above detailed model can be approximated by an effective model (Fig. 2c) described by the following two rate equations (See more detail in Supplementary Information Section 2): 
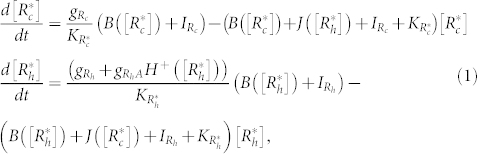
where 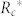 represents the active Rac1 (Rac1-GTP), and 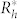 represents the active RhoA (RhoA-GTP). 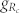
is the production rate for Rac1, 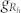 and 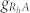 are basal and excitatory production rates for RhoA respectively. 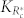 and 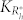 are their corresponding degradation rates. 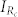 and 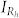 represent two external signals that drive the circuit, such as Grb2 and Gab1 in c-MET signaling. The Hill function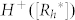 represents the transcriptional self-activation of RhoA. Two new functions, B and J, are defined to represent the GTP loading and hydrolysis rates (See details in Supplementary Information Section 2). The values of the both functions monotonically increase with an increase in the concentration of the active GTPases, as illustrated in Fig. 2d. The effective model was used for stability and bifurcation analysis while the detailed model was used for the dynamic simulations. The model derivation and the parameter estimations are described in details in the Supplementary Information Section 3.

### The Rac1/RhoA Circuit as a Three-way Switch

We started by analyzing the circuit dynamics in the absence of external signals (Grb2 = 0 and Gab1 = 0). As illustrated by a typical phase-plane diagram in Fig. 1c, Rac1/RhoA regulatory circuit can act as a three-way switch among the following three states: (high active RhoA/low active Rac1), (low active RhoA/high active Rac1), and (both high active RhoA and Rac1), which we denote as (1, 0), (0+, 1) and (1, 1) respectively. According to the experimental observations^9^,^23^,^24^,^25^,^40^, we associate the states (1, 0) and (0+, 1) with the amoeboid (A) and mesenchymal (M) phenotypes. Again, we used “0+” to denote some minimal level of active RhoA present in the rear end of mesenchymal cells and required for their individual migration^37^. Furthermore, we proposed to associate the (1, 1) state with the amoeboid-mesenchymal (A/M) hybrid phenotype that has been suggested in some recent experiments both for cancer cells and normal cells during early embryonic development^8^,^9^,^12^,^13^,^15^,^16^,^17^,^18^. Although these experiments lack quantitative measurement of the activity of Rac1 and RhoA, the mixed morphologies of these cells share the traits of both amoeboid and mesenchymal phenotypes. These properties may be indicative of relative high levels of both active RhoA and Rac1. Yet, it is clear that a direct measurement of these proteins is necessary to establish a direct association between these phenotypes and the model predicted hybrid phenotype.

Notably, for a small range of parameters, the Rac1/RhoA circuit can also act as a four-way switch, which gives rise to the coexistence of four states or quadra-stability (Supplementary Fig. S2a). The fourth state could be associated with the E/M hybrid phenotype that exhibits collective cell migration because activation of Rac1 and RhoA are needed for EMT^41^,^42^ and balanced, intermediate levels of Rac1 and RhoA activity are suggested in experiments on the E/M hybrid phenotype or partial EMT^43^,^44^,^45^. Since we mainly focused on solitary movement, we limited our analysis on the parameter range in which Rac1/RhoA circuit acts as a three-way switch.

### The Switch Response to External Activation and Inhibition Signals

**Figure 3:**
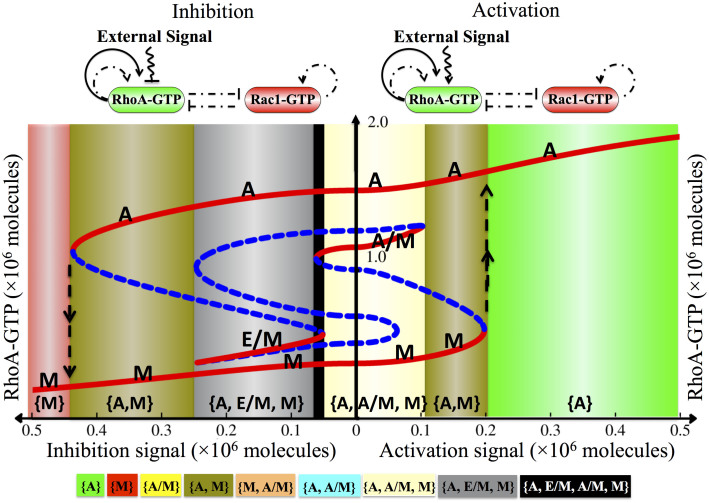
Bifurcation of RhoA-GTP protein levels in response to an external signal regulating the GTP loading rate of RhoA. The signal can either increase (activation) or decrease (inhibition) the GTP loading rate. The response to activation/inhibition is shown on the right/left side of the bifurcation respectively. The red solid lines indicate stable states and the blue dashed lines indicate unstable states. The bifurcation illustrates the possible coexistence (for some range of the signal) of four states: (*i*) the (1, 0) state with high RhoA-GTP and low Rac1-GTP, which corresponds to A phenotype; (*ii*) the (0+, 1), which corresponds to M phenotype; (*iii*) the (1, 1), which correspond to A/M phenotype; (*iv*) the (0, 0) state, which corresponds to E/M phenotype. The corresponding bifurcation of Rac1-GTP protein levels is shown in Supplementary Information Section 5. Co-existence of different phenotypes is associated with a multistable phase, highlighted by different background colors (see legend at the bottom). Starting from the (0+, 1) state (M phenotype, at bottom left part of the red curve), the system stays in the M phenotype when the inhibition signal is reduced; further switching the inhibition signal to an increasing activation signal leads the system to undergo a transition to the (1, 0) state (A phenotype, at top right part of the red curve). The transition is indicated by the dashed upward arrow at the boundary of the phase {A, M} and {A}. Similarly, increasing the inhibition signal can induce the transition from the (1, 0) state (A phenotype) back to the (0+, 1) state (M phenotype), as indicated by the downward arrow at the boundary of the phase {M} and {A, M}. Besides, cells may switch to the A/M or E/M phenotype due to noise in gene expression.

Next, we analyzed the response of the Rac1/RhoA circuit to an external input signal that drives the circuit through either Rac1 or RhoA. We modeled that the signal directly increases or decreases the basal GTP loading rates. In Fig. 3, we show the response to the external signal that affects the RhoA loading rate by the effective model shown in Fig. 2c. When the signal activates the basal GTP loading rate for RhoA, it gives rise to the coexistence of the diverse phenotypes A, M, A/M, all of which correspond to solitary movement. However, when the signal inhibits the loading rate, it also gives rise to collective cell migration of the E/M hybrid phenotype. As is also evident from Fig. 3, a high activation of the GTP loading rate leads the cell to a monostable phase {A} in which only amoeboid phenotype (A) exists, whereas low levels of GTP loading rate correspond to the monostable phase {M} in which only the mesenchymal phenotype (M) exists. The activity level of Rac1 for each phenotype is shown in Supplementary Fig. S3a. Our model is consistent with the experiments showing that cells with constitutively active RhoA are associated with amoeboid (A) or blebby amoeboid (BA) phenotype^46^; while cells with dominant negative RhoA usually exhibit a mesenchymal (M) phenotype^10^. It also predicted that, when the external signal acts only on RhoA, the induced transitions between A and M phenotypes are much easier than the transitions from A or M phenotype to A/M or E/M phenotype. This may explain why these hybrid phenotypes, A/M and E/M, were rarely observed during AMT in most of the experiments^14^.

We have shown the response to an external signal connected to Rac1 (see Supplementary Information Fig. S3b,c). In this case, inhibition of the Rac1 loading rate did not give rise to the existence of the E/M hybrid phenotype. However, this phenotype does exist when two external signals of inhibiting nature drive both Rac1 and RhoA simultaneously (Supplementary Fig. S3d). These results indicate that the cells may attain the E/M phenotype when GTP loading rates of RhoA or Rac1 are low (see blue area in Fig. 1a). However, when these GTP loading rates increase significantly due to the external signals, the cells are more likely to switch to one of the solitary modes of migration thus displaying reduced plasticity of spontaneous transitions among different migration phenotypes, which is consistent with experiments from Turner group^47^ (see green, red and yellow areas in Fig. 1a).

### The Switch Response to Input Signals from Grb2 and Gab1

As was mentioned earlier, Grb2 activates only Rac1, and Gab1 activates both Rac1 and RhoA. To understand the circuit response to these regulations, we first investigated the response of the circuit dynamics to either Grb2 or Gab1 (in terms of the corresponding bifurcation diagram) when they act individually. We found that when Grb2 level is increased, the cells adopt a mesenchymal (M) phenotype (Supplementary Fig. S4a,b); whereas Gab1 induces the cell to adopt an amoeboid (A) phenotype (Supplementary Fig. S4c,d). However, further high Gab1 signal can induce the cell to adopt the amoeboid/mesenchymal (A/M) phenotype since Gab1 can activate both Rac1 and RhoA (Supplementary Fig. S4e,f).

**Figure 4:**
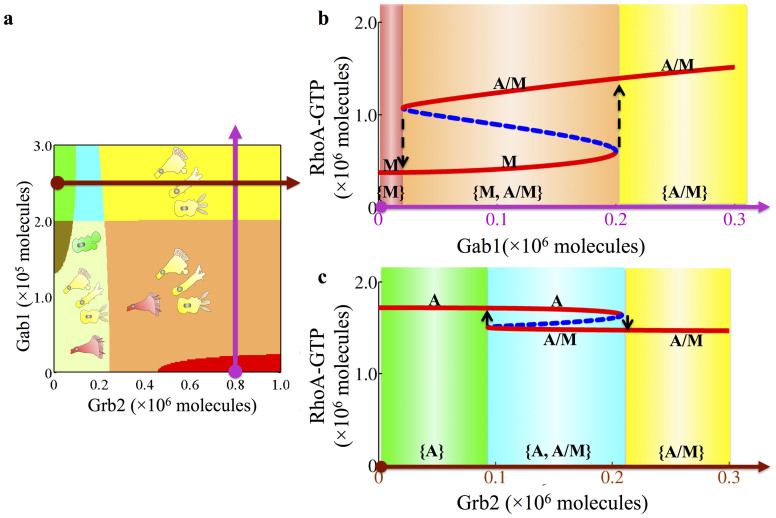
The circuit response to input signals from Grb2 and Gab1. (a) Two-parameter bifurcation phase diagram. Two input signals Grb2 (x-axis) and Gab1 (y-axis) are selected as the two control parameters. As explained in the text, each phase corresponds to a different combination of coexisting phenotypes (Phase plane diagrams for each phase are shown in Supplementary Fig. S5). For example, the orange area is the phase {M, A/M}, which means that cells in this phase can belong to either the M or the A/M phenotype. The colors used for different phases are explained by the legend in Fig. 3. (b) One-parameter bifurcation diagram for the circuit driven by Gab1 when the Grb2 level is fixed to be 8 × 10^5^ molecules. The corresponding trajectory in the two-parameter bifurcation phase diagram (Fig. 4a) is shown by a purple line. The transitions between the M and A/M phenotypes are illustrated by the dashed upward and downward arrows. (c) One-parameter bifurcation diagram for the circuit driven by Grb2 when the Gab1 level is fixed to be 2.5 × 10^5^ molecules. The corresponding trajectory in the two-parameter bifurcation phase diagram (Fig. 4a) is shown as a brown line. The transitions between the A and A/M phenotypes are illustrated by the upward and downward arrows.

Next, to understand the combined effect of Grb2 and Gab1, we constructed the two-parameter bifurcation diagram, as shown in Fig. 4a. Each phase corresponds to a particular situation in which one or several different phenotypes can coexist. More specifically, the possible phases are: 1. Phases with only one phenotype – {A}, {M} and {A/M}. 2. Phases in which two phenotypes can coexist – {A, A/M}, {M, A/M} and {A, M}. 3. A phase in which all three phenotypes coexistence – {A, M, A/M}. The detailed information of the various phases indicates the plasticity of cell migration as driven by different combinations of Grb2 and Gab1 signals. Depending on how Grb2 and Gab1 increase temporally, the cells follow different trajectories in this phase diagram and thus go through different phenotypic transitions as is illustrated in Fig. 4b and 4c.

The regulatory effect of Gab1 depends on the relative strengths of its activation to the GTP loading rates of Rac1 and RhoA (details are presented in the Supplementary Section 9). In the case of stronger activation of RhoA, Gab1 stimulates the cells to acquire the amoeboid phenotype. In contrast, in the case of stronger activation of Rac1, Gab1 stimulates the cells towards the A/M hybrid phenotype or the mesenchymal phenotype (Supplementary Fig. S7). Understanding the circuit response to input signal from Grb2 and Gab1 provides crucial clues regarding the pleiotropic effects of the c-MET pathway in promoting either the amoeboid or mesenchymal mode of migration and also transitions between them (AMT and MAT).

### Phenotypic Transitions Driven by the c-MET pathway

**Figure 5:**
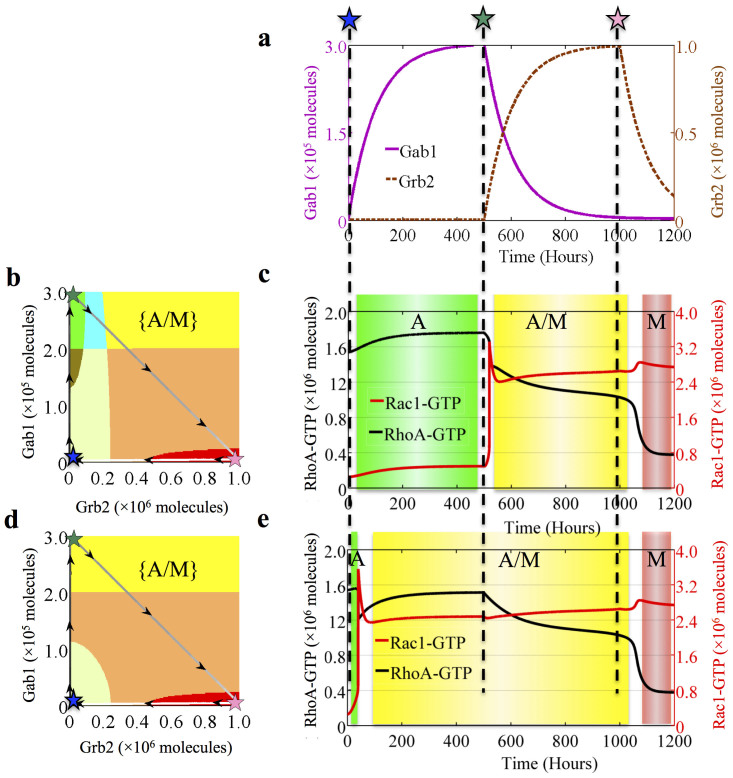
Temporal dynamics of the cells to HGF/SF treatment. (a) The time dependent Gab1 and the Grb2 signals that imitate the effect of HGF/SF treatment, as explained in the text. (b) Trajectory of the cell response projected on the phase diagram. The results are for a cell in which Gab1 activation of RhoA GTP loading is stronger than its activation of the Rac1 GTP loading (The parameters are *gtp*_*R_h_I*_2_ = 240*h*^−1^ and *gtp*_*R_c_I*_2_ = 90*h*^−1^ respectively). The solid line is the trajectory, and both the arrows on the line and the color gradient of the line (from black to white) indicate the time evolution. The blue star marks the initial condition, the green star marks the peak of Gab1 expression and the pink star marks the peak of Grb2 expression. (c) Time dynamics of the expression levels of Rac1-GTP (Red) and RhoA-GTP (Black) in response to the signals, Gab1 and Grb2. The different phenotypes during the transition are highlighted by different background colors, where green, yellow and red areas stand for A, A/M and M phenotype respectively. (d) Similar to (b) but for a cell in which Gab1 activation of Rac1 loading is stronger than its activation of the RhoA loading (The parameters are *gtp*_*R_c_I*_2_ = 1000*h*^−1^ and *gtp*_*R_h_I*_2_ = 240*h*^−1^ respectively). (e) Similar to (c) but for the case shown in (d).

To better understand the phenotypic transitions, we investigated the response dynamics of the Rac1/RhoA circuit when the input signals, Gab1 and Grb2, change in time. To recapitulate the possible response of the circuit to HGF/SF treatment, we chose time dependent functions for the levels of the Gab1 and Grb2 to mimic the cells' response to HGF/SF treatment (Fig. 5a). The HGF/SF signal leads to increase c-MET phosphorylation^26^, which in turn regulates Gab1 and Grb2 together with Met-Induced Mitochondrial Protein (Mimp) in a form of two coupled feed-forward loops (FFLs) as is shown in Fig. 1b^48^,^49^. Hence, the levels of Gab1 and Grb2 were modeled as two pulse signals with a time delay as shown in Fig. 5a^50^.

The form of the pulses is similar to that of a typical response of a FFL. Time delay is incorporated to reflect the effect of the feed-forward like coupling of Grb2 and Gab1 to c-MET (Gab1 responses ahead of Grb2). The simulated treatment starts with cell in the amoeboid (A) phenotype at the left bottom corner (shown as blue star) in the phase diagram shown in Fig. 5b. The cell stays in this phenotype when Gab1 is increased (Fig. 5c) but makes a transition into the hybrid (A/M) phenotype after Gab1 decreases and Grb2 increases, thus the activity of Rac1 increases while that of RhoA remains almost unchanged. Finally, after Grb2 also decreases, the cell goes through another transition from the hybrid (A/M) phenotype into the mesenchymal (M) one. The results illustrate how c-MET pathway can regulate the cells to switch between different migrating phenotypes. In Fig. 5d and 5e, we demonstrated the result for the same simulation but for a cell with different circuit parameters (a cell in which Gab1 activation of Rac1 GTP loading is stronger than its activation of the RhoA loading). In this case, the increase of Gab1 signal induces the cell to transit into the hybrid A/M phenotype instead of maintaining it in A phenotype. The different signal-response behaviors of these two simulations support that different cell lines (cells with different parameters) may respond differently to the regulatory signals Gab1 and Grb2.

The aforementioned results are consistent with experimental results and help to explain several experimental observations. In particular, c-MET pathway is reported to induce both the mesenchymal phenotype for several non-cancer and cancer cells^11^,^29^,^30^ and the amoeboid phenotype for some breast cancer cells^31^. Other experiments show that Grb2 is essential for TGF-  to induce a mesenchymal phenotype for some cancer cells^51^ and Gab1 can stimulate AMT by forming dorsal ruffles through its adaptor Nck^52^. These observations have been well captured by our simulations showing that mesenchymal phenotype can be induced either by Grb2 in one cell line or by Gab1 in another cell line with different parameters (see Fig. 5 and Supplementary Fig. S7).

### Testable Predictions

**Figure 6:**
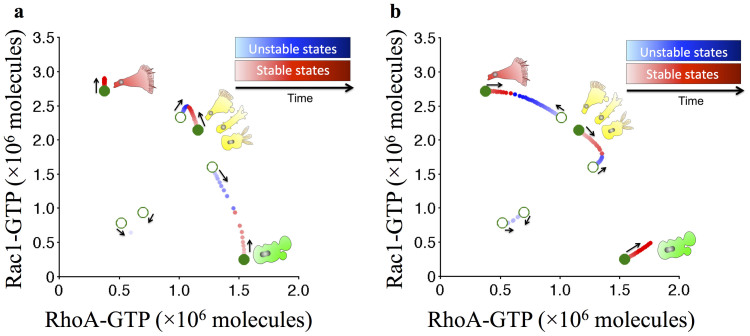
Circuit dynamics driven by the Grb2 and Gab1 signals shown in the Rac1/RhoA phase plane diagram. (a) The Grb2 signal increase from 0 to 1.0 × 10^6^ molecules (Gab1 = 0). Initially, the system is tristable (cells can be in either A, M or A/M phenotype). Next, Grb2 is gradually increased, during which we calculated a sequence of the stable states (The red dots that extend from the initial green stable states) and the saddle points among them (The blue dots that extend from the initial saddle points). In other words, the figure shows several superimposed phase plane diagrams for the cases of different Grb2 levels. When Grb2 is gradually increased, the A phenotype disappears first – the red and the blue dots come close and eventually disappear, implying that the cell loses the potential to be A phenotype. Upon further increase of Grb2, the A/M phenotype also disappears. It means that for high Grb2 input the cells can only be in the mesenchymal phenotype (See Supplementary Movie S1). (b) Similar to (a) but for an increase in Gab1 input from 0 to 3.0 × 10^5^ molecules (Grb2 = 0). In this case, the A/M phenotype is the first to disappear and then the mesenchymal phenotype. It means that for high Gab1 input the cells can only be in the amoeboid phenotype (See Supplementary Movie S2). The gradients of both red and blue from light to dark represent the temporal increase of Grb2 and Gab1 signals. The black arrows indicate the direction in which each state moves as the signal increases.

We present here our predictions that could be tested in future experiments. Input from Grb2 stimulates the cells towards mesenchymal phenotype (Fig. 6a) while input from Gab1 stimulates the cells towards the amoeboid phenotype (Fig. 6b). These predictions can be tested, in principle, by changing the expression of Grb2 and Gab1 in given cells. For a certain set of parameters, which can be considered representing some cell lines in which Gab1 has stronger activation of Rac1 GTP loading, Gab1 is predicted to stimulate the cells to become mesenchymal or the hybrid phenotype.

### Phenotype Distribution

Cells belonging to the same cell line often display non-heritable phenotypic variability^53^,^54^. Such variability can originate, for example, from local differences in the microenvironment (such as ECM rigidity) leading to differences in the circuit parameters of the individual cells. We have shown earlier that cells with different circuit parameters can respond in a different way to the input signals. Hence, we expect to see a distribution of phenotype for given input signals. As a first step to assess the expected nature of the population level distribution, we extended our simulations to a population of 5,000 cells, each with different circuit parameters. More specifically, the cells that compose the populations have ±5% variations from the original parameters.

**Figure 7:**
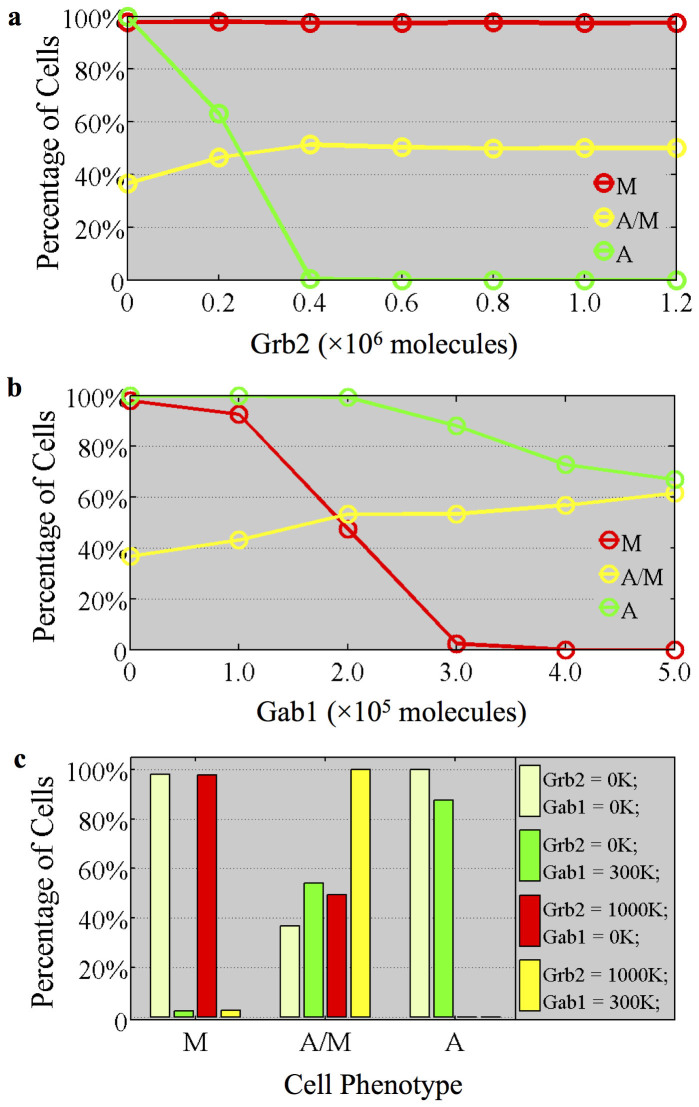
Phenotype distribution of a population of cells at different levels of Grb2 and Gab1 signals. (a) Phenotype distribution of a population of cells driven by Grb2 signal (Gab1 = 0). The cell parameters are randomly distributed over ±5% relative to the original parameters. The y-axis denotes the percentage of cells corresponding to a specific phenotype. The color of each line represents a phenotype. Under the stimulation of different level of Grb2, a population of cells has different phenotype distribution (percentage of cells shown in y-axis). For each point, the original phase of the cells depends on the level of Grb2 signal. Note that due to the co-existence of different phenotypes in one phase (e.g. in both the phases {A, M, A/M} and {A, M}; A and M phenotypes are present), the sum of the total percentages for one particular signal level can be more than 100%. For example, the initial phase with neither Gab1 nor Grb2 signals is {A, A/M, M}. About 100% cells can be A or M phenotype, and about 36% cells can be A/M. Some of these cells were in some multistable regime comprised of one or more of the phenotypes of A, M and A/M. (See Supplementary Fig. S8). (b) Phenotype distribution driven by Gab1 signal (Grb2 = 0). (c) Phenotype distribution under Grb2 and Gab1 regulations. The color of each bar, corresponding to the color definition above represents the initial cell phase. When both Grb2 and Gab1 signals are high, the cells are highly likely to be maintained in hybrid A/M phenotype.

In Fig. 7, we showed the percentages of cells that can be in one of the three different possible phenotypes, (A, A/M and M), for different levels of the input signals. We found that for high Grb2 or high Gab1 signal, a significant percentage of cells adopt the A/M phenotype (Fig. 7a,b). This result, which is obtained due to a weak robustness (high sensitivity to the circuit parameters), indicates that cells under high Grb2 or Gab1 signal are still sensitive to the conditions of their microenvironment. However, both high Grb2 and Gab1 signals can result in more cells being in the hybrid A/M phenotype (Fig. 7c). We also generated cell phase distribution for different initial conditions and found similar results (See Supplementary Fig. S8).

## Discussion

By considering the relationship between active levels of Rho GTPases and cell morphology, we were able to build a theoretical framework on Rac1/RhoA GTPase-based regulatory circuit to interpret some experimental observations about cancer cell migration phenotypes, and further present several testable predictions for future experiments.

In the current model, we devised the circuit to capture the GTP loading and hydrolysis based mechanism of mutual inhibition between the GTPases Rac1 and RhoA. As is showed in Supplementary Table S1, most of the connections have been discovered in breast cancer cells, but a few of them are found in the other cell lines. It is important to note that in some cell types, the circuit might be different; for instance, some positive feedback between Rac1 and RhoA is also reported in 3T3 fibroblasts via Dbs and mDia. However, this positive feedback seems to be cell-type specific and the fundamental mechanism still remains unknown^20^. Therefore, we assumed that the circuit we devised can be relevant to many types of cancers, and thereby can provide valuable understanding of the underlying mechanism of AMT in general.

We found the Rac1/RhoA regulatory circuit is usually a three-way switch. Such tristability has been shown to be a hallmark of many self-activating toggle switches (SATS), i.e. double negative feedback loops with self-activation on one or both of the elements of the loop^4^,^32^,^55^,^56^. Here, based on experimental observations^9^,^23^,^24^,^25^,^40^, we propose to associate the (1, 0) - high activity of RhoA but low activity of Rac1 state with the amoeboid phenotype, and to associate the (0+, 1) state - low activity of RhoA and high activity of Rac1 with the mesenchymal phenotype. Notably, it has been reported that high activity of RhoA can also inhibit cell migration by forming strong focal adhesions to ECM, that is specific to some microenvironments^35^ and cell types^36^. In the current model, we did not consider the interplay between cells and ECM. Hence, it is not possible to distinguish the migratory and stationary phenotypes in the case of high RhoA activity within the current theoretical framework presented here. The interaction between the Rac1-RhoA circuit and the ECM as mediated via integrins remains a subject for the future extension of the model.

In addition to the (1, 0) state (A phenotype) and the (0+, 1) state (M phenotype), the model predicts the existence of a third (1, 1) state with balanced relative high RhoA-GTP and Rac1-GTP. We proposed to associate this state with the experimentally observed^8^,^9^,^12^,^13^,^15^,^16^,^17^,^18^ hybrid ameboid/mesenchymal (A/M) phenotype. As was explained earlier, the reason for this association is as follows: both high RhoA-GTP and Rac1-GTP can enable cells in this state to obtain morphological properties resulting from both high actomyosin contractility and high actin polymerization, such as pseudopodal amoeboid phenotype mentioned above. Moreover, the downregulation of the effect of Rac1 or RhoA could induce the transition from A/M to canonical A or M phenotypes^8^,^13^,^16^. For example, the knockdown of RhoA or inactivation of myosin II switched cell migration from lobopodia (LP) or filopodia/blebs (LB) to exclusive lamellipodia (M) mode^13^,^16^. Since the (1, 1) state for different cell types might manifest itself as different phenotypes^8^,^9^,^12^,^13^,^15^,^16^,^17^,^18^, we lumped them together as A/M phenotypes in this work.

Regarding implications for cancer cell migration, we note that the cells belonging to the hybrid phenotype are expected to be more adaptable when cells migrate in a complex environment. More specifically, since these cells can display some properties of both amoeboid and mesenchymal phenotypes, and can also promote the transitions between these two phenotypes, they can more readily adopt to ECM with high variability. The model prediction presented here call for future direct measurements of both active levels of RhoA and Rac1 in these phenotypes to further prove our hypothetic association of the (1,1) state with these A/M phenotypes.

We also showed the diverse regulatory functions of Grb2 and Gab1 on Rac1/RhoA regulatory circuit. Since Grb2 and Gab1 are two different adaptors for c-MET receptor, their different functions in inducing cell migration may help explain how c-MET pathway can induce the cells to adopt either of these A or M phenotype, which are usually mutually exclusive. It may be noted that Grb2 and Gab1 both form Feed-Forward Loops (FFLs) with c-MET receptor, thus their activity may be separated in time. In Fig. 5, we show that AMT can be induced by sequential activation of Gab1 and Grb2 signals to the regulatory circuit. This indicates that integrating c-MET pathway in detail with Rac1/RhoA regulatory feedback loop in future might better explain the impact of HGF/SF on cell migration.

In this study, we have explored in detail the phenotypic plasticity pertaining to single cell migration. However, when sessile epithelial cells from primary carcinoma undergo EMT, they start moving collectively in the hybrid E/M state, and some of them further transit to move individually in mesenchymal or amoeboid mode; therefore either completing EMT^4^,^5^ or undergoing a Collective to Amoeboid Transition (CAT)^19^. The dynamic regulation between E, E/M, A, M and A/M phenotypes remains far from understood. Future investigations into understanding the coupled dynamics of EMT/MET and MAT/AMT regulatory networks – miR-200/ZEB and RhoA-Rac1 respectively – would be imperative in charting out the entire plasticity landscape that migrating carcinoma cells can adopt^5^.

As noted before, the current model does not incorporate intracellular diffusion of the GTPases, i.e. the effect of spatial distributions of these Rho GTPases. Since these effects can be significant, an important future extension of the model should be to incorporate the intracellular spatial dynamics of Rac1 and RhoA and its connection with the cell shape change between the three different phenotypes – A, M and A/M. More specifically, this would entail understanding the spatial distribution of these proteins within cells exhibiting different phenotypes, for instance, cells in M phenotype has high levels (1) of active Rac1 on the cell side extending the protrusion, and some minimal amount of active RhoA (0+) on the opposite pole, and the diffusion of molecules between different ends of the cell may introduce a time delay. The role of Cdc42, a Rho GTPase that is important for Rac1 localization and the formation of filopodia^37^,^57^, would also be decisive in this endeavor to understand how spatial distribution of activity levels of RhoA and Rac1 govern the cell morphology. Previous models for spatial segregation of Rho GTPases, based on reaction-diffusion mechanism, have explored the role of mutual inhibition and fast diffusion in stabilizing the cell shape^58^,^59^. A recent attempt in this direction used a simple Boolean model of RhoA and Rac1 activity in two different spatial compartments in the cell^60^. The Boolean framework considers the activity levels of RhoA and Rac1 to be binary - either ON (1) or OFF (0), and does not capture the existence of any intermediate state(s). However, we show that the Rac1/RhoA loop is a three-way switch that allows for the existence of hybrid A/M phenotypes. Therefore, our framework is better equipped to chart out comprehensively the shape space of a cell based on active levels of Rac1 and RhoA.

To conclude, our model provides a better understanding of the plasticity of cell migration and its regulation by external signals. Furthermore, it paves a promising way to understand how c-MET pathway is involved in carcinoma metastasis. Given the large number of current attempts to therapeutically target the c-MET pathway, understanding this relationship can provide some useful non-intuitive insights for therapeutic interventions to prevent metastasis.

## Author Contributions

B.H., M.L. and E.B.J. developed the theoretical framework. B.H. performed the simulations. B.H., M.L., M.K.J., I.T., J.O. and E.B.J. contributed to the analysis of the simulation results, comparison with experimental observations and the generation of the manuscript.

## Supplementary Material

Supplementary Information Supplementary Information

Supplementary Information Supplementary Movie S1

Supplementary Information Supplementary Movie S2
