# Supplementary material for: The three-way switch operation of Rac1/RhoA GTPase-based circuit controlling amoeboid-hybrid-mesenchymal transition
Source: Sci Rep. 2014 Sep 23;4:6449. doi: 10.1038/srep06449 (PMC4171704; doi:10.1038/srep06449)
Supplement: Supplementary Information [file srep06449-s1.pdf]

The three-way switch operation of Rac1/RhoA GTPase-based circuit controlling amoeboid-hybrid-mesenchymal transition

Bin Huang[1,2], Mingyang Lu[1], Mohit Kumar Jolly [1,3], Ilan Tsarfaty[5,*],
Jose' Onuchic [1,2,4,6,*], Eshel Ben-Jacob [1,6,7,*]

[1]Center for Theoretical Biological Physics, [2]Department of Chemistry, [3]Department of Bioengineering, [4]Department of Physics and Astronomy, [6]Department of Biosciences, Rice University, Houston, TX 77005-1827, USA
[5] Department of clinical microbiology and Immunology, Sackler School of Medicine
[7]School of Physics and Astronomy and The Sagol School of Neuroscience,
Tel-Aviv University, Tel-Aviv 69978, Israel

[*]Authors to whom correspondence should be sent: Ilan Tsarfaty Email: ilants@post.tau.ac.il; Jose' Onuchic Email: jonuchic@rice.edu; Eshel Ben-Jacob Email: eshelbj@gmail.com.

Supplementary Information

1. Rac1/RhoA Regulatory Circuits Construction

In the core regulatory circuit (Fig. 1b), Rac1-GTP and RhoA-GTP can mutually inactivate each other and also promote their own activation, thus introducing more nonlinearity in the circuit. Each of these regulations has been indicated experimentally, as shown in Supplementary Table S1. For most of them, they are discovered in breast cancer cells, but a few of them are found in the other cell lines. Here, we assumed that the circuit we built is conserved in most of human cancer cells. Therefore, the study of this circuit can provide some useful insights into amoeboid to mesenchymal transition for cancer cells in general.

Supplementary Table S1. Experimental evidences for Rac1/RhoA regulatory circuits.

Regulations	Involved Molecules*	References	Cell lines
Rac1-GTP inhibits RhoA-GTP	p190RhoGAP	Anjaruwee et al., 2003[1]<br>William et al., 2001[2]<br>Che-Hung et al., 2008[3]	Breast cancer cells,<br>Hela cells,<br>Rat1 fibroblasts
RhoA-GTP inhibits Rac1-GTP	FilGAP	Koji et al., 2012[4]	Breast, lung, prostate and colorectal adenocarcinoma cells
	ARHGAP22	Victoria et al., 2008[5]	Melanoma cells
Rac1-GTP self-activation	IRSp53/EP58	Yosuke et al., 2004[6]	Breast cancer cells,<br>Melanoma cells,<br>Fibrosarcoma cells et al.
	Hem-1	Orion et al., 2006[7]	Human neutrophil-like cells
RhoA-GTP self-activation	Dia1	Thomas et al., 2007[8]	Breast cancer cells, Human embryonic kidney cancer cell
RhoA transcriptional self-activation	c-Myc	Chia-Hsin et al., 2010[9]<br>Xose, 2010[10]	Breast cancer cells,<br>Fibroblasts
Grb2 activates Rac1-GTP	Ras	Esther et al., 2011[11]<br>John et al., 2002[12]<br>Peter et al., 1994[13]	Breast cancer cells,<br>Human embryonic kidney 293 cells et al.
Gab1 activates Rac1-GTP	Crk	Takuya et al., 2006[14]	Breast cancer cells,<br>Human synovial sarcoma cells
Gab1 activates RhoA-GTP	LARG	Dong et al., 2009[15]	Rat vascular smooth muscle cells

* Most regulations are mediated indirectly through GEFs or GAPs, such as p190RhoGAP.

To further clarify how the activity of Rac1 and RhoA are regulated by Gab1 and Grb2 signals, we constructed the connecting circuit as follow:

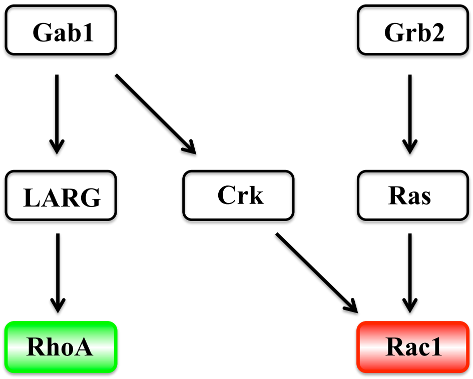

Supplementary Figure S1. The circuit connecting RhoA and Rac1 to Gab1 and Grb2 signals. Grb2 can accelerate the GTP loading rate of Rac1 through Ras with the help of Sos, PI3K. When Grb2 binds and activate SOS, Ras is then activated[11,13]. The activated Ras can activate Rac1 either in PI3K dependent pathway[11] or by Tiam1 in PI3K independent way[12]. Gab1 can accelerate the GTP loading rate of either Rac1 or RhoA through Crk/DOCK180[14] and LARG[15] respectively.

2. Theoretical framework for small GTPase-based Regulatory Circuits

To study the dynamics of Rac1/RhoA regulatory circuit, we developed a computational model for small *G*TPase-*b*ased Regulatory *C*ircuits (GBC). Here, we show in details how we derived the model from binding and unbinding reactions at molecular level. We first focused on the process of cycling for a typical Rho GTPase[16] (denoted as Rho) (Fig. 2a). The reactions for this process are

$$\text{Rho-DNA} \xrightarrow{k_1} \text{Rho} \qquad \text{SR1}$$

$$\text{Rho} + \text{GDP} \xrightarrow{k_2} \text{Rho-GDP} \qquad \text{SR2}$$

$$\text{Rho-GDP} + \text{GDI} \underset{k_4}{\overset{k_3}{\rightleftharpoons}} \text{Rho-GDI} \qquad \text{SR3}$$

$$\text{Rho-GDP} + \text{GTP} \xrightarrow{k_5} \text{Rho-GTP} + \text{GDP} \qquad \text{SR4}$$

$$\text{Rho-GDP} + \text{GTP} + \text{GEF} \xrightarrow{k_6} \text{Rho-GTP} + \text{GDP+GEF} \qquad \text{SR5}$$

$$\text{Rho-GTP} \xrightarrow{k_7} \text{Rho-GDP} + \text{Pi} \qquad \text{SR6}$$

$$\text{Rho-GTP} + \text{GAP} \xrightarrow{k_8} \text{Rho-GDP} + \text{Pi+GAP} \qquad \text{SR7}$$

$$\text{Rho-GDP} \xrightarrow{k_9} \text{Null} \qquad \text{SR8}$$

$$\text{Rho-GTP} \xrightarrow{k_{10}} \text{Null} \qquad \text{SR9}$$

where Rho-DNA represents the gene encoding this Rho GTPase, and Rho-GTP, Rho-GDP and Rho-GDI stand for Rho GTPase bound to GTP, GDP and GDI respectively. Reaction SR1 is for the production of Rho GTPase. Reaction SR2 represents our assumption that Rho protein first binds to GDP instead of GTP when it is translated.

Reaction SR3 is the reversible binding and releasing of GDI to Rho protein. Reaction SR4 is the intrinsic GTP loading process and Reaction SR5 is the activated GTP loading by GEFs. Reaction SR6 is the intrinsic GTP hydrolysis process and Reaction SR7 is the activated GTP hydrolysis by GAPs. At first, we did not consider the signals (I_1, I_2) that can regulate some GEFs or GAPs, thus [GEF] and [GAP] involved in the regulations of the GTP loading and hydrolysis are constant. Reaction SR8 and SR9 stand for the degradations of Rho-GDP and Rho-GTP, respectively. Since GDI is reported to stabilize Rho GTPases, we do not consider the degradation of Rho-GDI[17]. k_n (n = 1,2 …) is the reaction rate constant for each reaction. The model for these reactions can be given as:

$$
\begin{aligned}
\frac{d[R^f]}{dt} &= k_1 \bullet [R^g] - k_2 \bullet [GDP] \bullet [R^f] \\
\frac{d[R^I]}{dt} &= k_3 \bullet [GDI] \bullet [R] - k_4 \bullet [R^I] \\
\frac{d[R]}{dt} &= k_2 \bullet [GDP] \bullet [R^f] + k_4 \bullet [R^I] + k_7 \bullet [R^*] + k_8 \bullet [GAP] \bullet [R^*] \\
&\quad - k_3 \bullet [GDI] \bullet [R] - k_5 \bullet [GTP] \bullet [R] - k_6 \bullet [GTP] \bullet [GEF] \bullet [R] - k_9 \bullet [R] \\
\frac{d[R^*]}{dt} &= k_5 \bullet [GTP] \bullet [R] + k_6 \bullet [GTP] \bullet [GEF] \bullet [R] - k_7 \bullet [R^*] - k_8 \bullet [GAP] \bullet [R^*] - k_{10} \bullet [R^*]
\end{aligned}
\quad , (1)
$$

where R^f and R^g represent the Rho GTPase without binding to any molecules and the gene encoding Rho GTPase respectively. In order to simplify the equations, we proposed three assumptions here:

1) The levels of GDI, GTP and GDP are assumed to be constant, thus we can reduce some parameters by integrating the number of [GDI], [GTP] and [GDP] into the reaction rate constants (such as $k_3^{'} = k_3 \bullet [GDI]$).

2) The levels of each GEF and GAP are constant except the ones activated by the signals (I_1, I_2) we discussed later. Therefore, we can also integrate them into the reaction rate constants (such as $k_8^{'} = k_8 \bullet [GAP]$).

3) We assume that GDP is present in abundant amount. Rho can bind to GDP to produce inactive GDP-bound form (Rho-GDP) as soon as Rho protein is produced. Thus the $\frac{d[R^f]}{dt}$ equals to 0.

With these assumptions, the model can be converted to:

$$\frac{d[R^I]}{dt} = k_3^{'} \bullet [R] - k_4 \bullet [R^I]$$
$$\frac{d[R]}{dt} = G + k_4 \bullet [R^I] + (k_7 + k_8^{'}) \bullet [R^*]$$
$$- k_3^{'} \bullet [R] - (k_5^{'} + k_6^{'}) \bullet [R] - k_9 \bullet [R]$$
$$\frac{d[R^*]}{dt} = (k_5^{'} + k_6^{'}) \bullet [R] - (k_7 + k_8^{'}) \bullet [R^*] - k_{10} \bullet [R^*], \quad (2)$$

where $G = k_1 \bullet [R^g]$. $k_3^{'}$ and k_4 are binding and releasing rate constant for GDI respectively. k_7 and $k_5^{'}$ are intrinsic GTP hydrolysis and loading rate constants respectively, while $k_8^{'}$ and $k_6^{'}$ are activated GTP hydrolysis and loading rate constants due to GAPs and GEFs respectively.

Now we consider signals (I_1, I_2) in the GBC model, as shown in Fig. 2a. These signals can regulate the activity level of Rho GTPase through GEFs or GAPs. These signals can be external, such as Grb2 and Gab1 from c-Met pathway or other Rho GTPases like RhoA that can promotes the GTP hydrolysis of Rac1-GTP by activating its relevant GAPs; or internal such as the auto-regulations for Rac1 and RhoA. Therefore, the total GTP hydrolysis and loading rate constants (described by B and J functions respectively as given below) can be divided into three parts: the intrinsic ones, the activated ones by the GAPs and GEFs that are independent of the regulations of signals, and the activated

ones by the GAPs and GEFs that depend on the signals. The first two parts, which can be grouped as basal GTP hydrolysis and loading rate constants, are both constant. Yet, the latter activated rate constants (k_A) are related with the level of signals (I) and can be described by Hill function as:

$$H^+([I]) = \frac{[I]^n}{K_d^n + [I]^n} = \frac{k_A}{k_A^{\max}}, \ (3)$$

where $k_A^{\max}$ is the maximum activated rate constant. K_d is the dissociation constant which represents the threshold at which the activated rate (k_A) is at half maximum value. n is the Hill coefficient determining the steepness of the function. Thus, the activated GTP hydrolysis and loading rate constants by signal I_1 and I_2 can be expressed as $k_A^{GAP_{\max}} \bullet H^+([I_1])$ and $k_A^{GEF_{\max}} \bullet H^+([I_2])$, respectively. Considering these signals into the GBC model above (Supplementary Equation (2)), the signals-driven GBC model can be given as:

$$\begin{aligned}
\frac{d[R^I]}{dt} &= k_3^{'}\bullet[R] - k_4\bullet[R^I] \\
\frac{d[R]}{dt} &= G + k_4\bullet[R^I] + (k_7 + k_8^{''} + k_A^{GAP_{\max}}\bullet H^+([I_1]))\bullet[R^*] \\
&\quad - k_3^{'}\bullet[R] - (k_5^{'} + k_6^{''} + k_A^{GEF_{\max}}\bullet H^+([I_2]))\bullet[R] - k_9\bullet[R] \\
\frac{d[R^*]}{dt} &= (k_5^{'} + k_6^{''} + k_A^{GEF_{\max}}\bullet H^+([I_2]))\bullet[R] - (k_7 + k_8^{''} + k_A^{GAP_{\max}}\bullet H^+([I_1]))\bullet[R^*] - k_{10}\bullet[R^*]
\end{aligned} \quad , (4)$$

where we use $k_8^{''}$ and $k_6^{''}$ to represent the signal-independent activated rate constants for GTP hydrolysis and loading, respectively. To simplify Supplementary Equation (4), we defined two functions: one is B function standing for total GTP loading rate constant and the other is J function standing for total GTP hydrolysis rate constant. Both of them depend on the signals.

$$B([I_2]) = k_5^{'} + k_6^{''} + k_A^{GEF_{\max}} \bullet H^+([I_2])$$
$$J([I_1]) = k_7 + k_8^{''} + k_A^{GAP_{\max}} \bullet H^+([I_1]) \quad , (5)$$

Integrating Supplementary Equation (4) and (5), we can get a generic deterministic model (Supplementary Equation (6)) to describe the transition among GDI state, GDP state and GTP state of a typical Rho GTPase (Fig. 2a).

$$\frac{d[R^I]}{dt} = k_3^{'} \bullet [R] - k_4 \bullet [R^I]$$
$$\frac{d[R]}{dt} = G + k_4 \bullet [R^I] + J([I_1]) \bullet [R^*] - k_3^{'} \bullet [R] - B([I_2]) \bullet [R] - k_9 \bullet [R]$$
$$\frac{d[R^*]}{dt} = B([I_2]) \bullet [R] - J([I_1]) \bullet [R^*] - k_{10} \bullet [R^*] \quad , (6)$$

Utilizing above methods, we can deduce the deterministic model for the detailed circuit (Fig. 2), shown below:

$$\frac{d[R_c^I]}{dt} = gdi\_R_c \bullet [R_c] - dgdi\_R_c \bullet [R_c^I]$$
$$\frac{d[R_c]}{dt} = g_{R_c} + J([R_h^*]) \bullet [R_c^*] + dgdi\_R_c \bullet [R_c^I] - gdi\_R_c \bullet [R_c] - B([R_c^*]) \bullet [R_c] - I_{R_c} \bullet [R_c] - K_{R_c} \bullet [R_c]$$
$$\frac{d[R_c^*]}{dt} = B([R_c^*]) \bullet [R_c] + I_{R_c} \bullet [R_c] - J([R_h^*]) \bullet [R_c^*] - K_{R_c^*} \bullet [R_c^*]$$

$$\frac{d[R_h^I]}{dt} = gdi\_R_h \bullet [R_h] - dgdi\_R_h \bullet [R_h^I]$$
$$\frac{d[R_h]}{dt} = (g_{R_h} + g_{R_hA} \bullet H^+([R_h^*])) + J([R_c^*]) \bullet [R_h^*] + dgdi\_R_h \bullet [R_h^I] - gdi\_R_h \bullet [R_h] - B([R_h^*]) \bullet [R_h] - I_{R_h} \bullet [R_h] - K_{R_h} \bullet [R_h]$$
$$\frac{d[R_h^*]}{dt} = B([R_h^*]) \bullet [R_h] + I_{R_h} \bullet [R_h] - J([R_c^*]) \bullet [R_h^*] - K_{R_h^*} \bullet [R_h^*] \quad , (7)$$

where

$$
\begin{aligned}
&B([R_c^*]) = gtp\_R_c i + gtp\_R_c B + gtp\_R_c A \bullet H^+([R_c^*]) \\
&J([R_h^*]) = dgtp\_R_c i + dgtp\_R_c B + dgtp\_R_c A \bullet H^+([R_h^*]) \\
&I_{R_c} = gtp\_R_c I_1 \bullet H^+([I_1]) + gtp\_R_c I_2 \bullet H^+([I_2]) \\
\\
&B([R_h^*]) = gtp\_R_h i + gtp\_R_h B + gtp\_R_h A \bullet H^+([R_h^*]) \\
&J([R_c^*]) = dgtp\_R_h i + dgtp\_R_h B + dgtp\_R_h A \bullet H^+([R_c^*]) \\
&I_{R_h} = gtp\_R_h I_2 \bullet H^+([I_2])
\end{aligned}
\quad , (8)
$$

Here, R_c^I, R_c, R_c^* stand for Rac1 in GDI-bound state, GDP-bound state, and GTP-bound state, respectively, R_h^I, R_h, R_h^* stand for the different states for RhoA. B function is the total GTP loading rate constant, which contains the intrinsic GTP loading rate constant ($gtp\_R_c i$ and $gtp\_R_h i$), the activated GTP loading rate constant ($gtp\_R_c B$ and $gtp\_R_h B$) by GEFs not involved in the auto-regulations and the activated GTP loading rate constant ($gtp\_R_c A$ and $gtp\_R_h A$) resulting from auto-regulations. J function is the total GTP hydrolysis rate constant, which also contains the intrinsic GTP hydrolysis rate constant ($dgtp\_R_c i$ and $dgtp\_R_h i$), the activated GTP hydrolysis rate constant ($dgtp\_R_c B$ and $dgtp\_R_h B$) by GAPs not involved in the mutually regulations and the activated GTP hydrolysis rate constant ($dgtp\_R_c A$ and $dgtp\_R_h A$) resulting from the mutual regulations. gdi_R_c, $dgdi\_R_c$, gdi_R_h, and $dgdi\_R_h$ are the binding and unbinding rate constants for GDI. There are two external signals here: Grb2 (I_1) and Gab1 (I_2). I_{R_c} is the activated GTP loading rate constant for Rac1 by both Grb2 ($gtp\_R_c I_1$) and Gab1 ($gtp\_R_c I_2$) signals, and I_{R_h} is the activated GTP loading rate constant for RhoA by Gab1 ($gtp\_R_h I_2$) signal.

As the GTP-bound states of RhoA and Rac1 are the only active regulators of cell migration, we focus on the concentration of Rac1-GTP and RhoA-GTP and reduce the equations to two coupled equations for R_c^* and R_h^*. Assuming the total expression of Rac1 and RhoA reach equilibrium with their degradation, therefore

$$\begin{aligned}\frac{d[R_{ctot}]}{dt} = \frac{d[R_c^I]}{dt} + \frac{d[R_c]}{dt} + \frac{d[R_c^*]}{dt} = 0 \\ \frac{d[R_{htot}]}{dt} = \frac{d[R_h^I]}{dt} + \frac{d[R_h]}{dt} + \frac{d[R_h^*]}{dt} = 0\end{aligned} \quad , (9)$$

Then we can get

$$g_{R_c} - K_{R_c} \bullet [R_c] - K_{R_c^*} \bullet [R_c^*] = 0$$

$$(g_{R_h} + g_{R_hA} \bullet H^+([R_h^*])) - K_{R_h} \bullet [R_h] - K_{R_h^*} \bullet [R_h^*] = 0 \quad , (10)$$

If K_{R_c} (K_{R_h}) is same as $K_{R_c^*}$ ($K_{R_h^*}$), $[R_c]$ and $[R_h]$ can be expressed as a function of $[R_c^*]$ and $[R_h^*]$:

$$[R_c] = \frac{g_{R_c}}{K_{R_c^*}} - [R_c^*]$$

$$[R_h] = \frac{(g_{R_h} + g_{R_hA} \bullet H^+([R_h^*]))}{K_{R_h^*}} - [R_h^*] \quad , (11)$$

By substituting these expressions back into Supplementary Equation (7), we simplified the original model to four equations as below:

$$\frac{d[R_c^I]}{dt} = gdi\_R_c \bullet [R_c] - dgdi\_R_c \bullet [R_c^I]$$

$$\frac{d[R_c^*]}{dt} = \frac{g_{R_c}}{K_{R_c^*}} \bullet (B([R_c^*]) + I_{R_c}) - (B([R_c^*]) + J([R_h^*]) + I_{R_c} + K_{R_c^*}) \bullet [R_c^*]$$

$$\frac{d[R_h^I]}{dt} = gdi\_R_h \bullet [R_h] - dgdi\_R_h \bullet [R_h^I]$$

$$\frac{d[R_h^*]}{dt} = \frac{(g_{R_h} + g_{R_hA} \bullet H^+([R_h^*]))}{K_{R_h^*}} \bullet (B([R_h^*]) + I_{R_h}) - (B([R_h^*]) + J([R_c^*]) + I_{R_h} + K_{R_h^*}) \bullet [R_h^*], \quad (12)$$

Since only active Rac1 and RhoA cause downstream reactions, we can focus on the two-coupled equations with variable $[R_c^*]$ and $[R_h^*]$ as an effective model to approximate the detail model.

$$\frac{d[R_c^*]}{dt} = \frac{g_{R_c}}{K_{R_c^*}} \bullet (B([R_c^*]) + I_{R_c}) - (B([R_c^*]) + J([R_h^*]) + I_{R_c} + K_{R_c^*}) \bullet [R_c^*]$$

$$\frac{d[R_h^*]}{dt} = \frac{(g_{R_h} + g_{R_hA} \bullet H^+([R_h^*]))}{K_{R_h^*}} \bullet (B([R_h^*]) + I_{R_h}) - (B([R_h^*]) + J([R_c^*]) + I_{R_h} + K_{R_h^*}) \bullet [R_h^*] \quad , (13)$$

where the first part such as $\frac{g_{R_c}}{K_{R_c^*}} \bullet (B([R_c^*]) + I_{R_c})$ is the effective production rate, while the second part such as $(B([R_c^*]) + J([R_h^*]) + I_{R_c} + K_{R_c^*}) \bullet [R_c^*]$ is the effective degradation part. Notice that the self-activation on GTP loading process can activate both the effective production rate and the effective degradation rate. These simplified equations describe the interaction between active Rac1 and RhoA more clearly, and can be used to determine the stability of Rac1/RhoA regulatory circuit.

3. Parameters estimation

The values of most of the parameters considered in our model are not known exactly, but we hereby explain how we estimated the parameter values.

The degradation rates are selected due to the half-lives of each molecule from experiments. The half-life for Rac1 is about 2 hours[18], while the half-life of RhoA is about 5 hours[19]. Thus, we set their degradation rate to be 0.1 h^{-1}.

The total amount of Rac1 and RhoA protein are respectively 82-123 ng/10^6 cells and 38-75 ng/10^6 cells. These values are averaged from two epithelial cell lines, MDCK and ECV[20]. MDCK and ECV have similar amount of Rac1 and RhoA, where MDCK cells have about 56±14 ng/10^6 cells RhoA and 124±27 ng/10^6 cells Rac1 while ECV304 cells have about 50±15 ng/10^6 cells RhoA and 82±14 ng/10^6 cells Rac1. We used the average values of them here to ensure validity of our parameters. Since the molecular weight of these small G-proteins is about 22 kDa (3.65×10^{-11}ng), the molecular numbers of Rac1 and RhoA per cell are 2.25×10^6 -3.37×10^6 molecules and 1.04×10^6-2.05×10^6 molecules, respectively [20]. Thus, we set the basal transcriptional rates for Rac1 (g_{R_c}) and RhoA (g_{R_h}) to 3.40×10^5 molecules/hour and 1.60×10^5 molecules/hour. Also, due to the transcriptional self-activation by RhoA, we assume that for the cells in amoeboid state, they can have high expression of RhoA similar to that of Rac1 in cells with mesenchymal morphology. Thus, the activated transcriptional rate (g_{R_hA}) for RhoA is also set to 3.40×10^5 molecules/hour.

GTPases have a slow intrinsic GTP hydrolysis and loading ability. Experiments show that the intrinsic GTP hydrolysis rates for Rac1 and RhoA are about 6.6 h^{-1} and 1.32 h^{-1}, respectively[21,22]. GAPs can accelerate the hydrolysis rates by 5 to 4000 folds[22,23]. Thus we set $dgtp\_R_cB$ to 110 h^{-1} and $dgtp\_R_cA$ to 200 h^{-1} for Rac1, and set $dgtp\_R_hB$ to 311 h^{-1} and $dgtp\_R_hA$ to 88 h^{-1} for RhoA. For GTP loading process, the intrinsic rates are both about 0.54 h^{-1} for active Rac1 and RhoA[24]. Experiments show that GEFs can accelerate the rates by 3.5 to 1000 folds[24,25,26]. Similarly, we set gtp_R_cB to 20 h^{-1} and gtp_R_cA to 530 h^{-1} for Rac1, and set gtp_R_hB to 110 h^{-1} and gtp_R_hA to 196 h^{-1} for RhoA. Also, we take 2000 h^{-1} for both GDI binding (gdi_R_c and gdi_R_h) and releasing ($dgdi\_R_c$ and $dgdi\_R_h$) rate constants because Rac1 was observed to stay on the membrane for 2s[27].

In addition, since regulations such as the auto-regulations and the mutual inhibitions between Rac1-GTP and RhoA-GTP are indirect, we considered Hill function coefficients to be 4. Also, for Grb2 and Gab1 signals, we integrated them in the model using hill functions. Since their concentration is suggested to be about 1μM[28], namely about 1×10^6 molecules by considering the typical diameter of a eukaryotic cell to be 10μm, we set their corresponding hill function thresholds ($Tsd\_gtp\_R_cI_1$, $Tsd\_gtp\_R_hI_2$ and $Tsd\_gtp\_R_cI_2$) to 5×10^5 molecules. Since we integrated these signals into our model by hill functions, it is worthy to note that it is the ratio between the concentration of Gab1 and Grb2 to their thresholds instead of their absolute concentration values that actually affects the effects of these signals. Thus, the absolute values we used for Gab1 and Grb2 signals can always be adopted by modifying the threshold values.

Supplementary Table S2. Parameters for Rac1/RhoA regulatory Circuit

Parameters	Value	Unit	Description
Rac1			
g_{R_c}	3.4×10^5	molecules/h	Production rate
K_{R_c}	0.1	h^{-1}	Degradation rate for Rac1-GDP
$K_{R_c^*}$	0.1	h^{-1}	Degradation rate for Rac1-GTP
gdi_R_c	2.0×10^3	h^{-1}	Binding rate for GDI to Rac1-GDP
$dgdi\_R_c$	2.0×10^3	h^{-1}	Dissociation rate for Rac1-GDI
$gtp\_R_c i$	0.54	h^{-1}	Intrinsic GTP loading rate
$gtp\_R_c B$	19.46	h^{-1}	Activated GTP loading rate
$gtp\_R_c A$	530	h^{-1}	Activated GTP loading rate by self-activation
$gtp\_R_c A_0$	1.9×10^6	molecules	Threshold for self-activation
$n_{gtp\_R_c A}$	4	-	Hill coefficient for self-activation
$dgtp\_R_c i$	6.6	h^{-1}	Intrinsic GTP hydrolysis rate
$dgtp\_R_c B$	105.4	h^{-1}	Activated GTP hydrolysis rate
$dgtp\_R_c A$	198	h^{-1}	Activated GTP hydrolysis rate by RhoA
$dgtp\_R_c A_0$	1.2×10^6	molecules	Threshold for RhoA inactivation
$n_{dgtp\_R_c A}$	4	-	Hill coefficient for RhoA inactivation
RhoA			
g_{R_h}	1.6×10^5	molecules/h	Basal production rate
$g_{R_h A}$	3.4×10^5	molecules/h	Excitatory production rate
$g_{R_h A_0}$	8.0×10^5	molecules	Threshold for transcriptionally self-activation
$n_{g_{R_h A}}$	4	-	Hill coefficient for self-activation
K_{R_h}	0.1	h^{-1}	Degradation rate for RhoA-GDP
$K_{R_h^*}$	0.1	h^{-1}	Degradation rate for RhoA-GTP

gdi_R_h	2.0×10^3	h^{-1}	Binding rate for GDI to RhoA-GDP
$dgdi\_R_h$	2.0×10^3	h^{-1}	Dissociation rate for RhoA-GDI
$gtp\_R_h i$	0.54	h^{-1}	Intrinsic GTP loading rate
$gtp\_R_h B$	109.46	h^{-1}	Activated GTP loading rate
$gtp\_R_h A$	196	h^{-1}	Activated GTP loading rate by self-activation
$gtp\_R_h A_0$	1.0×10^6	molecules	Threshold for self-activation
$n_{gtp\_R_h A}$	4	-	Hill coefficient for self-activation
$dgtp\_R_h i$	1.32	h^{-1}	Intrinsic GTP hydrolysis rate
$dgtp\_R_h B$	308.68	h^{-1}	Activated GTP hydrolysis rate
$dgtp\_R_h A$	89	h^{-1}	Activated GTP hydrolysis rate by Rac1
$dgtp\_R_h A_0$	1.3×10^6	molecules	Threshold for Rac1 inactivation
$n_{dgtp\_R_h A}$	4	-	Hill coefficient for Rac1 inactivation
Signals			
$gtp\_R_c I_1$	240	h^{-1}	Activated GTP loading rate for Rac1 by Grb2
$gtp\_R_c I_{10}$	5.0×10^5	molecules	Threshold for Grb2 activation on Rac1
$n_{gtp\_R_c I_1}$	2	-	Hill coefficient for Grb2 activation on Rac1
$gtp\_R_h I_2$	240	h^{-1}	Activated GTP loading rate for RhoA by Gab1
$gtp\_R_h I_{20}$	5.0×10^5	molecules	Threshold for Gab1 activation on RhoA
$n_{gtp\_R_h I_2}$	2	-	Hill coefficient for Gab1 activation on RhoA
$gtp\_R_c I_2$	90	h^{-1}	Activated GTP loading rate for Rac1 by Gab1
$gtp\_R_c I_{20}$	5.0×10^5	molecules	Threshold for Gab1 activation on Rac1
$n_{gtp\_R_c I_2}$	2	-	Hill coefficient for Gab1 activation on Rac1

4. The roles of auto-regulations on Rac1 and RhoA

As showed in Fig.1c, Rac1/RhoA regulatory circuit usually can be a three-way switch (Tristability). In a narrow range of parameters, this circuit can even be quadra-stable (Supplementary Fig. S2a). This multistability are mainly due to the auto-regulations in each side of the circuit, which can increase the nonlinearity in the system and open up additional stable steady state(s)[29]. Without these auto-regulations, only bistability can be found at most (Supplementary Fig. S2b). Yet, having the auto-regulation on at least one of the GTPases possibly makes the circuit tristable (Supplementary Fig. S2c,b). Thus, the auto-regulations in this circuit are essential to the multistable behavior.

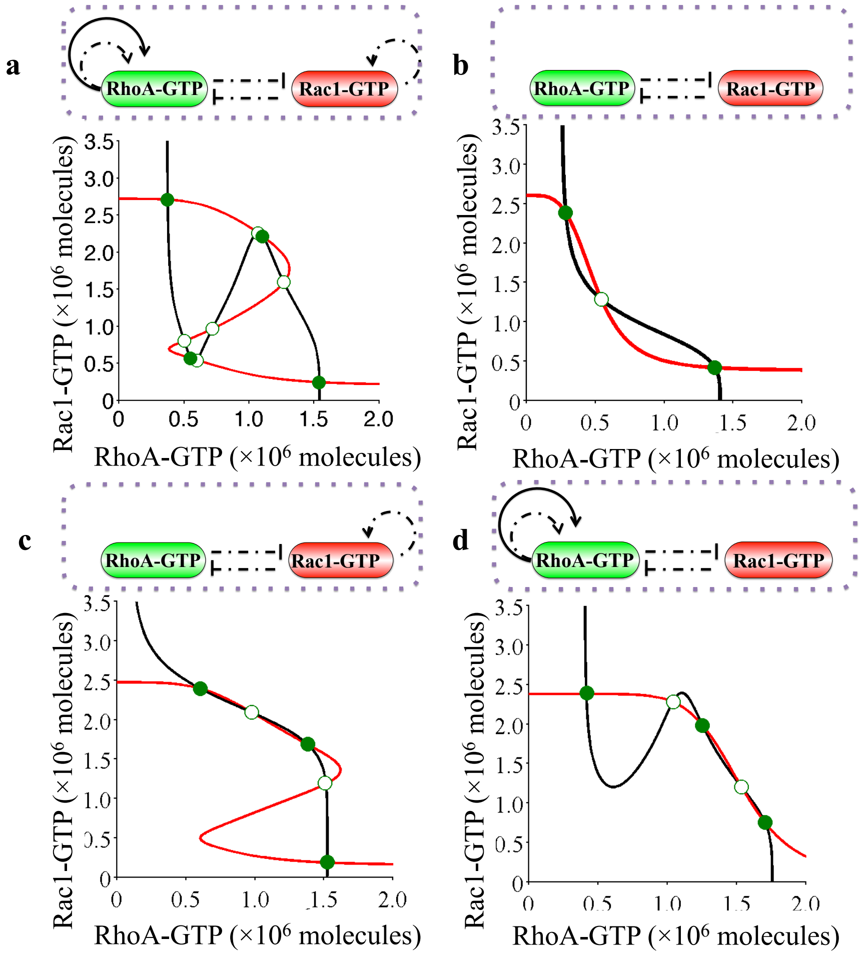

Supplementary Figure S2. Effect of auto-regulations of GTPases on the multistability of the circuit. Nullclines are plotted for the circuit with auto-regulations on both GTPases **(a)**, the circuit without auto-regulations **(b)** and the circuit with auto-regulation only on RhoA **(c)** or Rac1 **(d)**.

5. Respond of Rac1/RhoA regulatory circuit to Activation or Inhibition External Signals.

Here we explore the circuit with the external signal on Rac1. When the signal is of activating nature (I_2 is set to be positive), we observed all phenotypes that undergo individual migration – A, M, A/M. High level of this signal lead the circuit to momostable phase ({M}) with mesenchymal phenotype (M). When the signal is of inhibiting nature (I_2 is set to be negative), we still observe individual migration phenotypes instead of the E/M phenotype. Also, at high level of this signal, this circuit still display bistability with A and M phenotypes (Supplementary Fig. S3b,c).

Furthermore, we had external signals on both Rac1 and RhoA and showed the phase diagram for these two signals. When one of these signal is of activating nature, we rarely observed E/M phenotype. However, when both of them are of inhibiting nature, E/M phenotype is observed for a large range of parameters. This implies that at different values of external signals, this circuit might connect the collective migration (E/M) with individual migration modes (A, M and A/M).

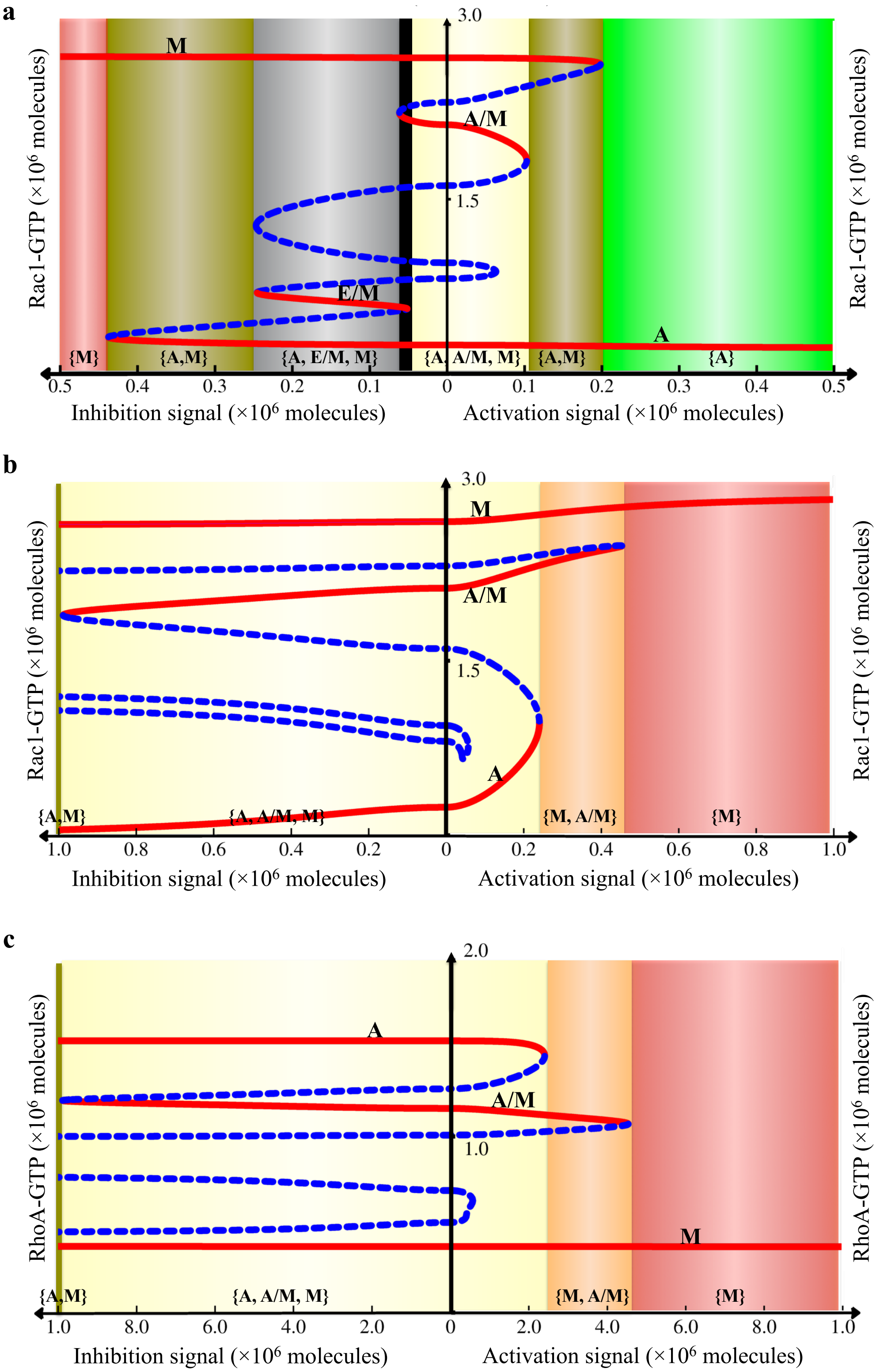
a
Rac1-GTP (×10⁶ molecules)
3.0
1.5
M
A/M
E/M
A
{M}
{A,M}
{A, E/M, M}
{A, A/M, M}
{A,M}
{A}
0.5 0.4 0.3 0.2 0.1 0 0.1 0.2 0.3 0.4 0.5
Inhibition signal (×10⁶ molecules)
Activation signal (×10⁶ molecules)
b
Rac1-GTP (×10⁶ molecules)
3.0
1.5
M
A/M
A
{A,M}
{A, A/M, M}
{M, A/M}
{M}
1.0 0.8 0.6 0.4 0.2 0 0.2 0.4 0.6 0.8 1.0
Inhibition signal (×10⁶ molecules)
Activation signal (×10⁶ molecules)
c
RhoA-GTP (×10⁶ molecules)
2.0
1.0
A
A/M
M
{A,M}
{A, A/M, M}
{M, A/M}
{M}
1.0 8.0 6.0 4.0 2.0 0 2.0 4.0 6.0 8.0 1.0
Inhibition signal (×10⁶ molecules)
Activation signal (×10⁶ molecules)

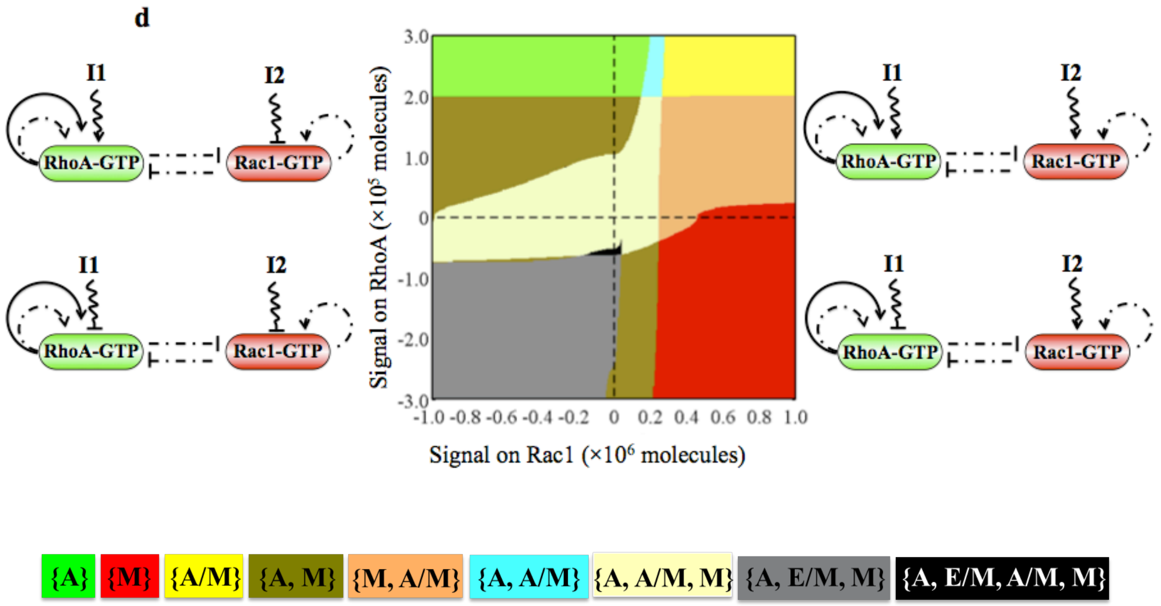

Supplementary Figure S3. Bifurcations and phase diagram of the circuit when driven by external signals. **(a)** The complementary bifurcation for Fig. 3 in main article. Here it is showed in the term of Rac1-GTP protein level. **(b)** Bifurcation of Rac1-GTP protein levels when driven by external signal on Rac1. **(c)** Bifurcation of RhoA-GTP protein levels when driven by external signal on Rac1. The red solid line stands for stable states and the blue dash line stands for unstable states. **(d)** Phase diagram of external signals both on RhoA (I_1) and Rac1 (I_2). The positive values stand for activation signals, while the negative values represent the inhibition signals. The circuit for each quadrant is showed beside it. Different coexistences (Phases) are highlighted by different background colors, as illustrated at bottom.

6. Bifurcation diagrams for the circuit driven by Grb2 and Gab1 signals.

Here, we calculated the one-parameter bifurcation diagrams for this regulatory circuit driven only by Grb2 signal or Gab1 signal. In each diagram, the increasing signals finally drive the cells to mono-stable phases, namely M or A phenotype. Since Gab1 activate both Rac1 and RhoA, when Gab1 increases further, the cells finally are induced to A/M phenotype (Supplementary Fig. S4e,f).

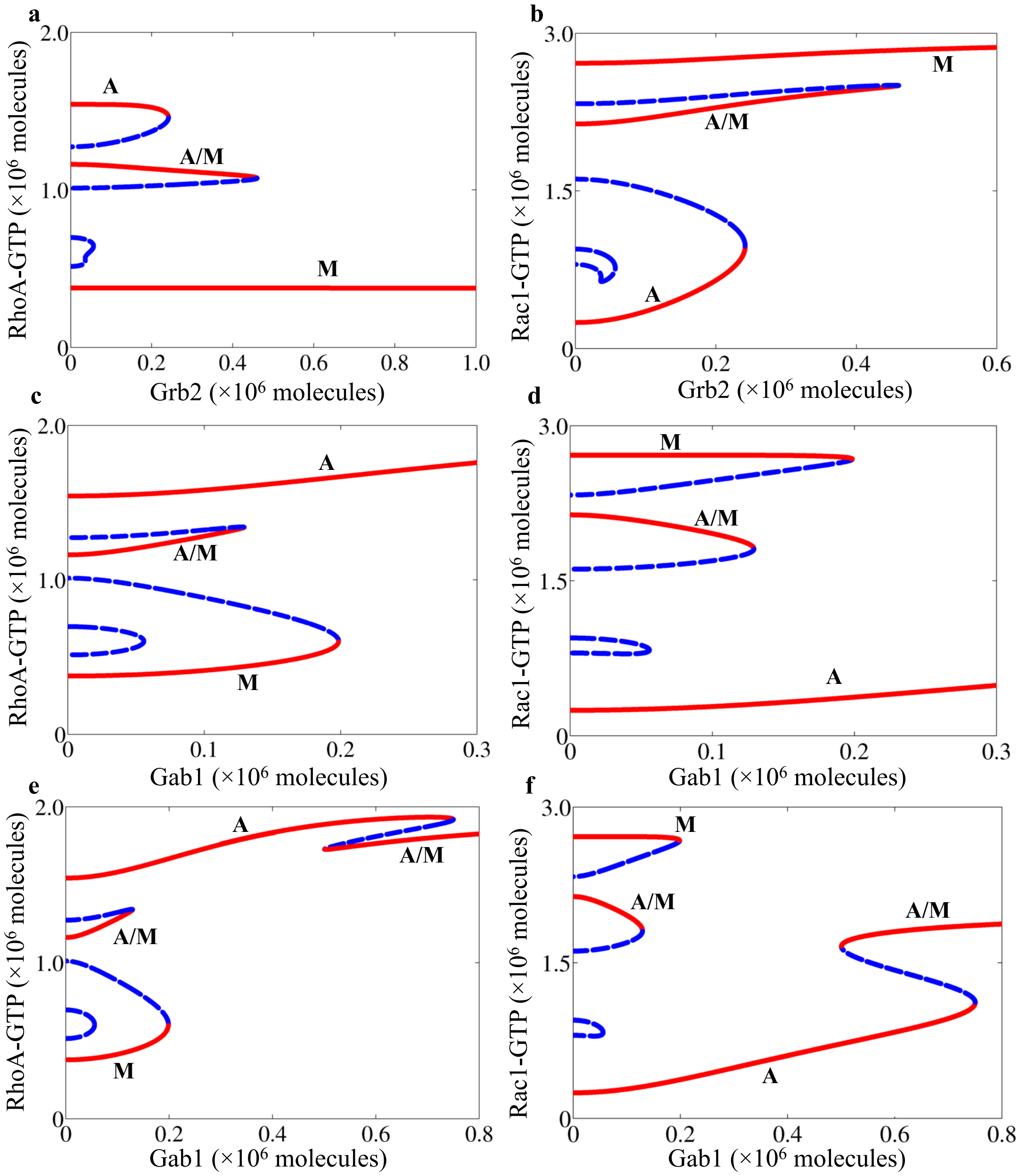

Supplementary Figure S4. One-parameter bifurcation diagrams for Grb2 and Gab1 regulation. **(a)** Bifurcation diagrams as the function of Grb2 and Rac1-GTP. **(b)** Bifurcation diagrams as the function of Grb2 and RhoA-GTP. **(c)** Bifurcation diagrams as the function of Gab1 and Rac1-GTP. **(d)** Bifurcation diagrams as the function of Gab1 and RhoA-GTP. When Gab1 signal increase further, the cells finally are induced to A/M phenotype (**e, f**). Blue dashed lines stand for the unstable states, while red solid lines stand for the stable states. The phenotypes corresponding to each stable line are labeled in the figures.

7. Phase plates for different phases in Grb2/Gab1 phase diagram

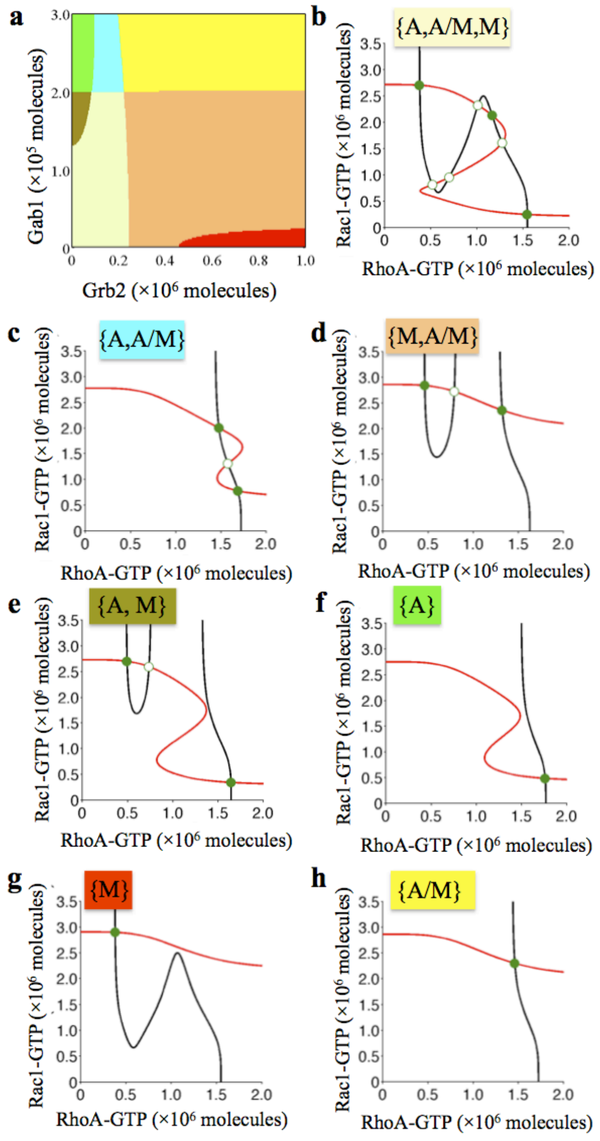

Supplementary Figure S5. Details about the different phases in Grb2/Gab1 phase diagram for Rac1/RhoA regulatory circuit. **(a)** Phase diagram using Grb2 and Gab1 signals as two parameters (also in Fig. 4a in the main text). **(b)** to **(h)** show the nullclines for the circuit at different phases - one phase for tri-stability **(b)**, three phases for bi-stability **(c, d, e)** and three phases for mono-stability **(f, g, h)**.

8. Respond of Rac1/RhoA regulatory circuit to Grb2 and Gab1 signals

As showed in Fig. 4, Grb2 signal can lead the cells to M phenotype while Gab1 signal can lead the cell to A phenotype. Differing Grb2 and Gab1 signals can drive cell to follow different trajectories in the phase diagram (Supplementary Fig. S6a) and go through different phenotypic transitions. Here we present the complementary bifurcations (Supplementary Fig. S6b,c) for Fig. 4. There bifurcations are showed in term of the level of Rac1-GTP instead of RhoA-GTP.

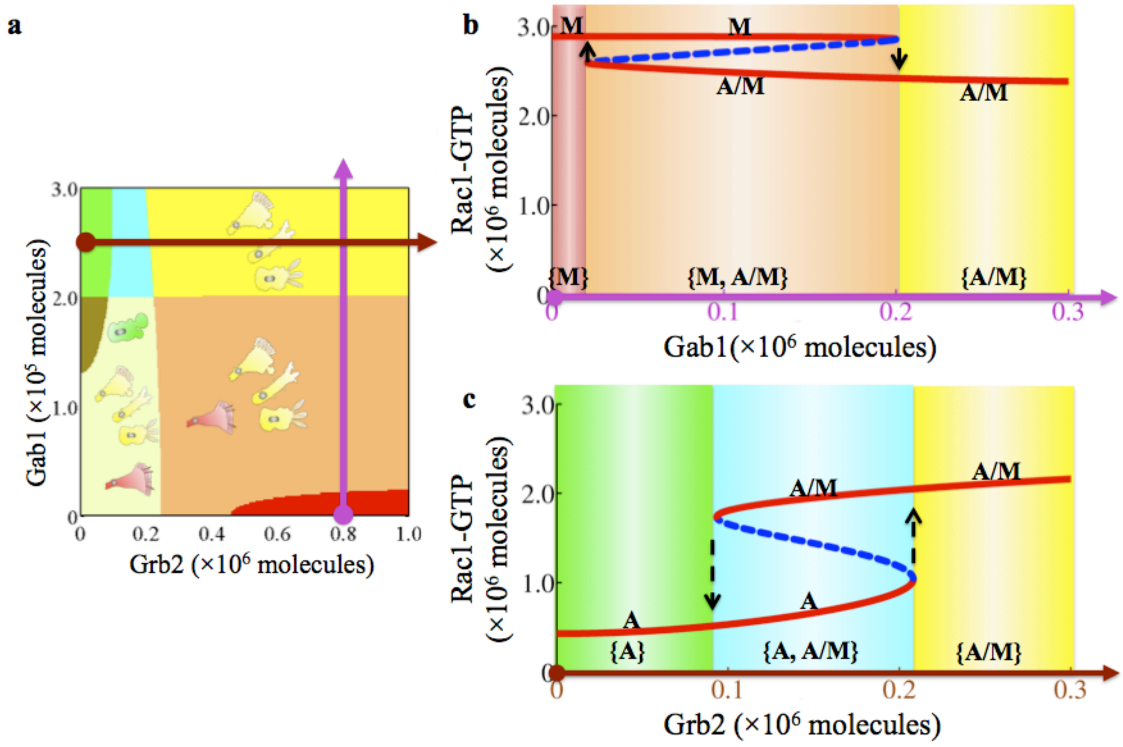

Supplementary Figure S6. Phase-diagram and bifurcations of the driven Rac1/RhoA regulatory circuit. **(a)** The phase diagram of the circuit driven by Grb2 and Gab1 signals simultaneously. **(b)** Bifurcation of the circuit driven by Gab1 signal when Grb2 signal is fixed at 8×10^5 molecules. **(c)** Bifurcation of the circuit driven by Grb2 signal when Gab1 signal is fixed at 2.5×10^5 molecules. These bifurcations are complementary to the bifurcations in Fig. 4, and are showed in term of the level of Rac1-GTP instead of RhoA-GTP.

9. Different activation strength on Rac1 and RhoA by Gab1 signal

Gab1 signal can activate the GTP loading for both Rac1 and RhoA. While selecting the model parameters, we set the activation of RhoA by Gab1 to be stronger than that of Rac1. This was motivated by preliminary observations (Tsarfaty unpublished) in which overexpression of Gab1 renders the cell to obtain the amoeboid phenotype. However, for some cell lines, it may not be this case. To test the effect of changing the relative strength of Gab1 activation, we fixed the activated GTP loading rate constant for RhoA ($gtp\_R_hI_2$) to be 240 h^{-1} and change the rate constant for Rac1 ($gtp\_R_cI_2$) from 90 h^{-1} to 1000 h^{-1} (Supplementary Fig. S4b). Compared with the phase diagrams from current model (Supplementary Fig. S4a), we found the area denoting the {A/M, M} and {A/M} phases expand with an increase in $gtp\_R_cI_2$, while the area for the mono-phenotypic phases for A phenotype shrinks and even disappears. If we keep $gtp\_R_cI_2$ unchanged as 1000 h^{-1} and reduce $gtp\_R_hI_2$ to 90 h^{-1}, namely Gab1's activation on RhoA is attenuated, the regions of existence for both {A/M, M} and {M} phases further expand (Supplementary Fig. S4c). In other words, for cell lines in which Gab1's activation on

Rac1 is stronger than its activation on RhoA, cells would most probably be seen in M or A/M phenotype instead of A phenotype on the overexpression of Gab1.

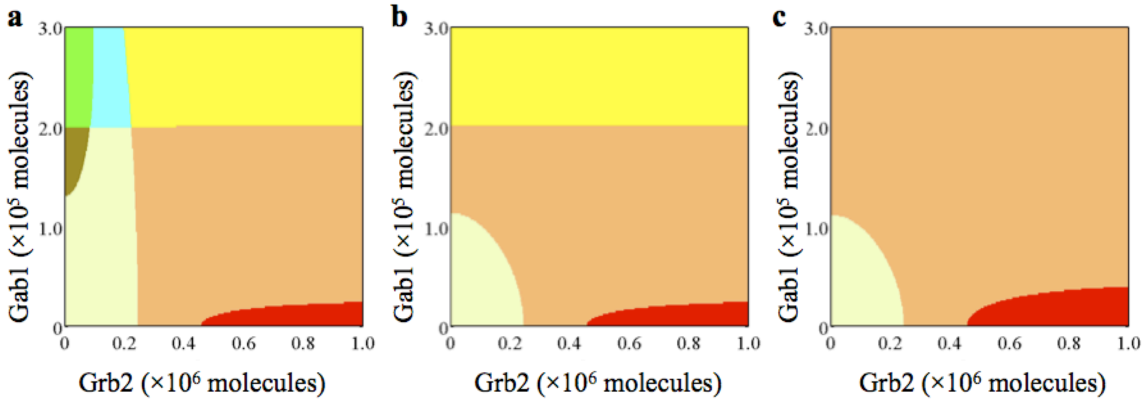

Supplementary Figure S7. Phase diagrams for different activation strength of Gab1 on Rac1. **(a)** The current model, whose stimulated GTP loading rate of Rac1 ($gtp\_R_cI_2$) by Gab1 is 90 h^{-1}. **(b)** The stimulated GTP loading rate of Rac1 ($gtp\_R_cI_2$) by Gab1 is 1000 h^{-1}, and other parameters are same as current model. **(c)** The stimulated GTP loading rate ($gtp\_R_cI_2$) of Rac1 by Gab1 is 1000 h^{-1} and that of RhoA ($gtp\_R_hI_2$) by Gab1 is 90 h^{-1}, and other parameters are same as current model.

10. Phase distribution on a population of cells.

In the main text, Fig. 8 showed the phenotype distribution of a population of 5,000 cells when parameters are randomized in ±5% range and the RhoA/Rac1 system is being regulated by Grb2 and/or Gab1. Here, we have calculated their phase distribution. Supplementary Fig. S8 shows the percentages of cells that can be present in one of the 7 different possible phases for different levels of the input signals. In the case in which the Rac1/RhoA circuit receives only the Grb2 signals or only the Gab1 signals (Supplementary Fig. S8a,b), we can still observe a significant percentage of cells in other phases, such as {M, A/M}, instead of the expected {M} or {A} alone, which indicates

the sensitivity of the cells to the change of parameters. However, both high Grb2 and Gab1 signals can result in more cells being in the {A/M} (Supplementary Fig. S8c).

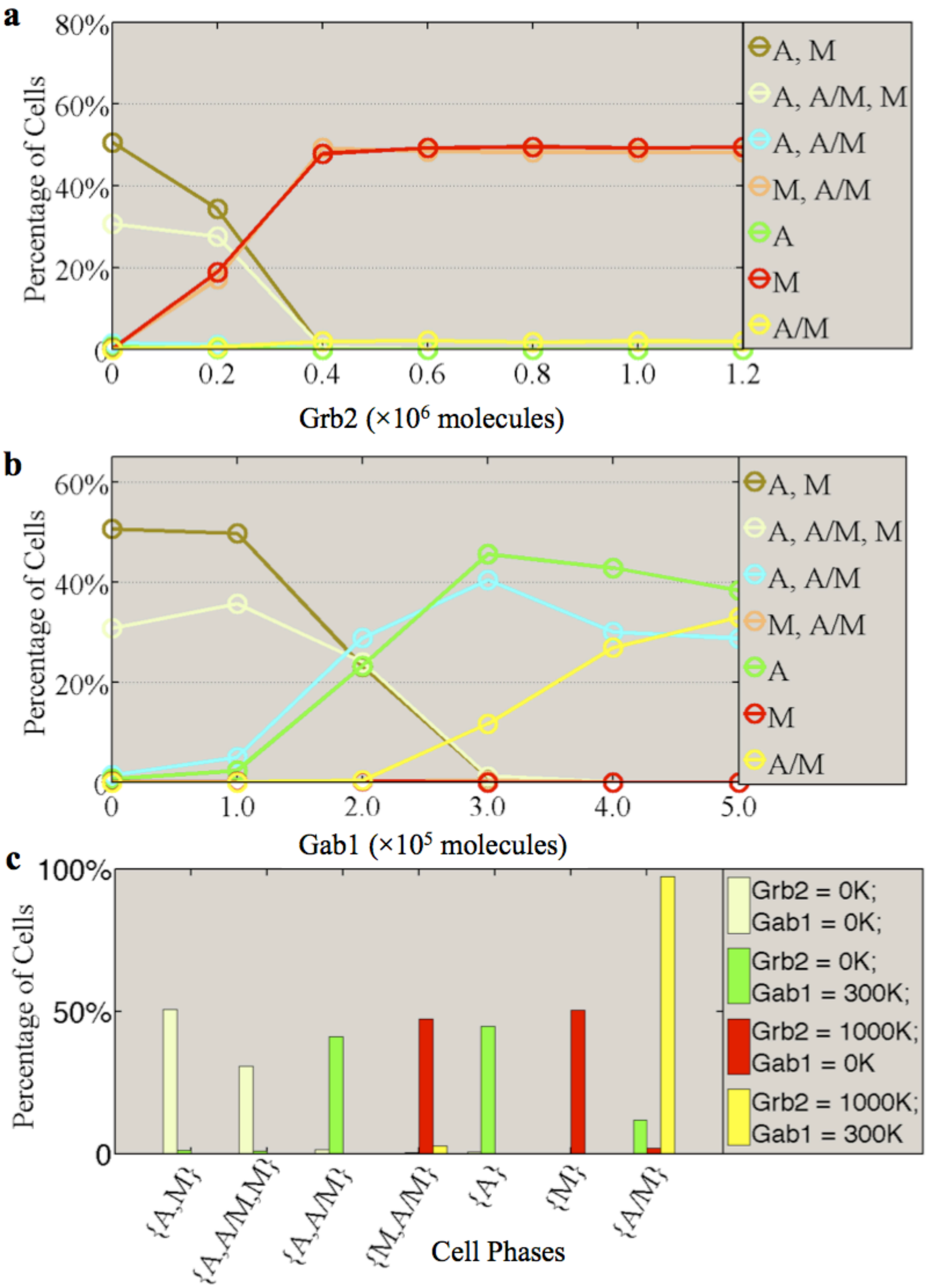

Supplementary Figure S8. (a) Phase distribution of a population of cells with parameters being randomized in the range of ±5% driven by Grb2 signal (Gab1 = 0). The

color of each line represents different phase. The original phase of the cells for each point depends on the level of Grb2 signal. **(b)** Phase distribution driven by Gab1 signal (Grb2 = 0). **(c)** Phase distribution under Grb2 and Gab1 regulations. The color of each bar corresponding to the color definition above represents the original cell phase.

References

1. Nimnual, A. S., Taylor, L. J. & Bar-Sagi, D. Redox-dependent downregulation of Rho by Rac. *Nat. Cell Biol.* **5,** 236–241 (2003).
2. Arthur, W. T. & Burridge, K. RhoA inactivation by p190RhoGAP regulates cell spreading and migration by promoting membrane protrusion and polarity. *Mol. Biol. Cell* **12,** 2711–2720 (2001).
3. Shen, C.-H. *et al.* Breast tumor kinase phosphorylates p190RhoGAP to regulate rho and ras and promote breast carcinoma growth, migration, and invasion. *Cancer Res.* **68,** 7779–7787 (2008).
4. Saito, K., Ozawa, Y., Hibino, K. & Ohta, Y. FilGAP, a Rho/Rho-associated protein kinase–regulated GTPase-activating protein for Rac, controls tumor cell migration. *Mol. Biol. Cell* **23,** 4739–4750 (2012).
5. Sanz-Moreno, V. *et al.* Rac Activation and Inactivation Control Plasticity of Tumor Cell Movement. *Cell* **135,** 510–523 (2008).
6. Funato, Y., Terabayashi, T. & Suenaga, N. IRSp53/Eps8 Complex Is Important for Positive Regulation of Rac and Cancer Cell Motility/Invasiveness. *Cancer Res* (2004).
7. Weiner, O. D. *et al.* Hem-1 Complexes Are Essential for Rac Activation, Actin Polymerization, and Myosin Regulation during Neutrophil Chemotaxis. *PLoS Biol.* **4,** e38 (2006).
8. Kitzing, T. M. *et al.* Positive feedback between Dia1, LARG, and RhoA regulates cell morphology and invasion. *Genes Dev.* **21,** 1478–1483 (2007).
9. Chan, C.-H. *et al.* Deciphering the transcriptional complex critical for RhoA gene expression and cancer metastasis. *Nat. Cell Biol.* **12,** 457–467 (2010).
10. Bustelo, X. R. A transcriptional cross-talk between RhoA and c-Myc inhibits the RhoA/Rock-dependent cytoskeleton. *Landes Biosci.* (2010). doi:10.4161/sgtp.1.1.12986
11. Castellano, E. & Downward, J. RAS Interaction with PI3K: More Than Just Another Effector Pathway. *Genes Cancer* **2,** 261–274 (2011).
12. Lambert, J. M. *et al.* Tiam1 mediates Ras activation of Rac by a PI(3)K-independent mechanism. *Nat. Cell Biol.* (2002). doi:10.1038/ncb833
13. Janes, P. W., Daly, R. J., deFazio, A. & Sutherland, R. L. Activation of the Ras signalling pathway in human breast cancer cells overexpressing erbB-2. *Oncogene* **9,** 3601–3608 (1994).
14. Watanabe, T. *et al.* Adaptor Molecule Crk Is Required for Sustained Phosphorylation of Grb2-Associated Binder 1 and Hepatocyte Growth Factor–

Induced Cell Motility of Human Synovial Sarcoma Cell Lines. *Mol. Cancer Res.* **4,** 499–510 (2006).
15. Wang, D. *et al.* A Role for Gab1/SHP2 in Thrombin Activation of PAK1 Gene Transfer of Kinase-Dead PAK1 Inhibits Injury-Induced Restenosis. *Circ. Res.* **104,** 1066–1075 (2009).
16. Jaffe, A. B. & Hall, A. Rho GTPases: biochemistry and biology. *Annu Rev Cell Dev Biol* **21,** 247–269 (2005).
17. Boulter, E. *et al.* Regulation of Rho GTPase crosstalk, degradation and activity by RhoGDI1. *Nat. Cell Biol.* **12,** 477–483 (2010).
18. Oberoi, T. K. *et al.* IAPs regulate the plasticity of cell migration by directly targeting Rac1 for degradation. *EMBO J.* **31,** 14–28 (2011).
19. Rolli-Derkinderen, M. Phosphorylation of Serine 188 Protects RhoA from Ubiquitin/Proteasome-Mediated Degradation in Vascular Smooth Muscle Cells. *Circ. Res.* **96,** 1152–1160 (2005).
20. Michaelson, D. *et al.* Differential localization of Rho GTPases in live cells regulation by hypervariable regions and RhoGDI binding. *J. Cell Biol.* **152,** 111–126 (2001).
21. Fiegen, D. Alternative Splicing of Rac1 Generates Rac1b, a Self-activating GTPase. *J. Biol. Chem.* **279,** 4743–4749 (2003).
22. Zhang, B. & Zheng, Y. Regulation of RhoA GTP hydrolysis by the GTPase-activating proteins p190, p50RhoGAP, Bcr, and 3BP-1. *Biochemistry (Mosc.)* **37,** 5249–5257 (1998).
23. Scheffzek, K. & Ahmadian, M. R. GTPase activating proteins: structural and functional insights 18 years after discovery. *Cell. Mol. Life Sci. CMLS* **62,** 3014–3038 (2005).
24. Gasmi-Seabrook, G. M. C. *et al.* Real-time NMR Study of Guanine Nucleotide Exchange and Activation of RhoA by PDZ-RhoGEF. *J. Biol. Chem.* **285,** 5137–5145 (2009).
25. Wu, X., Ramachandran, S., Lin, M. J., Cerione, R. A. & Erickson, J. W. A Minimal Rac Activation Domain in the Unconventional Guanine Nucleotide Exchange Factor Dock180. *Biochemistry (Mosc.)* **50,** 1070–1080 (2011).
26. Bourguignon, L. Y., Zhu, H., Shao, L. & Chen, Y. W. Ankyrin-Tiam1 interaction promotes Rac1 signaling and metastatic breast tumor cell invasion and migration. *J. Cell Biol.* **150,** 177–191 (2000).
27. Sako, Y. *et al.* Single-Molecule Imaging of Signaling Molecules in Living Cells. *Single Mol.* **1,** 159–163 (2000).
28. Carlier, M.-F. *et al.* GRB2 Links Signaling to Actin Assembly by Enhancing Interaction of Neural Wiskott-Aldrich Syndrome Protein (N-WASp) with Actin-related Protein (ARP2/3) Complex. *J. Biol. Chem.* **275,** 21946–21952 (2000).
29. Lu, M. *et al.* Tristability in Cancer-Associated MicroRNA-TF Chimera Toggle Switch. *J. Phys. Chem. B* **117,** 13164–13174 (2013).
